# Integrative phenotyping of glycemic responders upon clinical weight loss using multi-omics

**DOI:** 10.1038/s41598-020-65936-8

**Published:** 2020-06-08

**Authors:** Armand Valsesia, Anirikh Chakrabarti, Jörg Hager, Dominique Langin, Wim H. M. Saris, Arne Astrup, Ellen E. Blaak, Nathalie Viguerie, Mojgan Masoodi

**Affiliations:** 1 Nestlé Institute of Health Sciences, Lausanne, Switzerland; 2 INSERM, UMR 1048, Institute of Metabolic and Cardiovascular Diseases, Toulouse, France; 3 University of Toulouse, Paul Sabatier University, Toulouse, France; 4 Toulouse University Hospitals, Laboratory of Clinical Biochemistry, Toulouse, France; 5 Department of Human Biology, NUTRIM, School of Nutrition and Translational Research in Metabolism, Maastricht University Medical Centre+(MUMC+), Maastricht, The Netherlands; 6 University of Copenhagen, Department of Nutrition, Exercise and Sports, Faculty of Science, Copenhagen, Denmark; 7 Institute of Clinical Chemistry, Inselspital, Bern University Hospital, Bern, Switzerland

**Keywords:** Computational biology and bioinformatics, Biomarkers, Endocrinology, Cardiovascular diseases, Endocrine system and metabolic diseases, Metabolic disorders

## Abstract

Weight loss aims to improve glycemic control in obese but strong variability is observed. Using a multi-omics approach, we investigated differences between 174 responders and 201 non-responders, that had lost >8% body weight following a low-caloric diet (LCD, 800 kcal/d for 8 weeks). The two groups were comparable at baseline for body composition, glycemic control, adipose tissue transcriptomics and plasma ketone bodies. But they differed significantly in their response to LCD, including improvements in visceral fat, overall insulin resistance (IR) and tissue-specific IR. Transcriptomics analyses found down-regulation in key lipogenic genes (e.g. *SCD*, *ELOVL5*) in responders relative to non-responders; metabolomics showed increase in ketone bodies; while proteomics revealed differences in lipoproteins. Findings were consistent between genders; with women displaying smaller improvements owing to a better baseline metabolic condition. Integrative analyses identified a plasma omics model that was able to predict non-responders with strong performance (on a testing dataset, the Receiving Operating Curve Area Under the Curve (ROC AUC) was 75% with 95% Confidence Intervals (CI) [67%, 83%]). This model was based on baseline parameters without the need for intrusive measurements and outperformed clinical models (p = 0.00075, with a +14% difference on the ROC AUCs). Our approach document differences between responders and non-responders, with strong contributions from liver and adipose tissues. Differences may be due to de novo lipogenesis, keto-metabolism and lipoprotein metabolism. These findings are useful for clinical practice to better characterize non-responders both prior and during weight loss.

## Introduction

Obesity is a major risk factor for a number of co-morbidities including cardiovascular disease, dyslipidemia, hypertension, insulin resistance, type-2 diabetes (T2D), non-alcoholic fatty liver (NAFLD) and cancer^1–4^.

Weight loss can be achieved with energy-restricted dietary interventions and generally leads to improved glycemic control^5–7^. Recent evidence shows that low-caloric diet interventions can enable T2D remission and have superior efficacy compared to routine primary care^7^. Nevertheless, large inter-individual variability is observed in LCD studies regarding the capacity in particular to maintain weight loss^8–10^. A key outcome of caloric restriction, apart from desired reduction in fat mass, is the effect on adipose tissue function^9,11^. Adipose tissue is important in energy homeostasis and responding dynamically to caloric intake^12–14^. This is, partly due to lipolysis rate and adipose tissue triacylglycerol (TAG) turnover in response to caloric intake. The adipose tissue expandability hypothesis suggests that insulin resistance is caused by lipotoxicity resulting from adipose tissue inability to expand further, leading to ectopic deposition of lipids in non-adipose organs such as liver, muscle and pancreatic beta cells^15^. Comprehensive assessment of circulating lipid pattern could be a useful marker of insulin resistance and it may reflect adipose tissue dysfunction and hepatic *de novo* lipogenesis.

We were the first to report that even within subjects achieving significant weight loss (>8% of initial body mass), only a fraction would achieve significant glycemic improvements^16^. Specifically, subjects could be classified as glycemic responders and non-responders based on a lipid signature reflecting changes following LCD. Interestingly, these subjects only appeared to differ in their response to LCD whereas they had similar baseline body weight and glycemic variables. However, the underlying mechanism and physiological changes are not fully understood.

Considering the complexity of defining mechanism of action in a clinical cohort, we thus sought to perform a deeper characterization of these subjects using additional omics datasets including metabolomics and proteomics (>1,100 proteins) of plasma, together with transcriptomics (RNA-sequencing) of adipose Tissue (AT) biopsies. We also aimed to investigate further the tissue specific insulin resistance to clarify the contribution of different organs in the obese subjects following LCD intervention.

In this report, we acquired and analyzed multi-omics datasets from the Diet, Obesity and Genes (DiOGenes) study, one of the largest weight maintenance intervention of its kind^17^. We studied differences between subjects previously characterized as glycemic responders and non-responders^16^. We further investigated their molecular and physiological differences both at baseline and during weight loss intervention.

## Material/Subjects and Methods

### Study design

**Figure 1:**
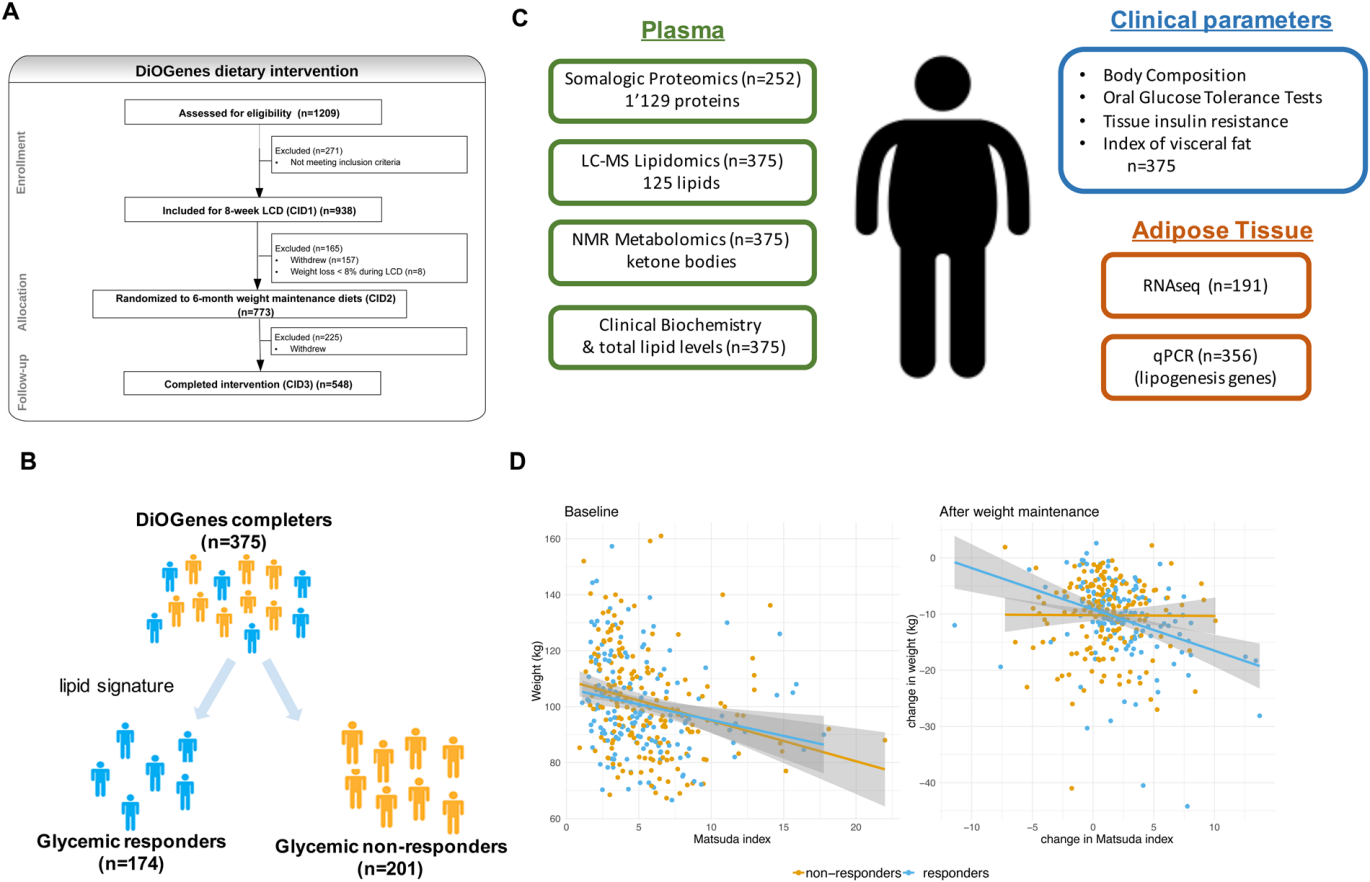
Flowchart for DiOGenes clinical intervention and omics analyses. (**A**) Clinical intervention with the number of participants entering the different phases as well as drop-outs are indicated. (**B**) Stratification into responders/non-responders (**C**) overview of omics datasets (all data available before and after LCD) (**D**) weight and glycemic characteristics at baseline and upon LCD for responders and non-responders. Abbreviations: CID, Clinical Intervention Day; LCD, low-caloric diet; QC: Quality Control; scAT: sub-cutaneous Adipose Tissue biopsies; WMD, Weight Maintenance Diets.

DiOGenes was a multicenter, randomized controlled dietary intervention study, involving eight European countries^17,18^ (ClinicalTrials.gov number, NCT00390637). The study has been described in detail previously^16,18^ and a CONSORT diagram of the clinical intervention is presented in Fig. 1A. Briefly, 938 overweight/obese, non-diabetic, adults (Body Mass Index (BMI) between 27 and 45 kg/m^2^, blood fasting glucose below 6.1 mmol/L) underwent an 8-week weight-loss phase using a complete meal replacement low calorie diet (LCD). The LCD provided 800 kcal/day (Modifast, Nutrition et Santé France). Among the 781 participants who completed the LCD, 773 achieved >8% weight loss and were randomized to a 26-week weight maintenance diet. A total of 548 subjects completed the Weight Maintenance Diet (WMD), among which 375 (~70%) had available qc-ed plasma samples at all intervention time-points: baseline (Clinical Intervention Day 1, CID1), after 8-week of LCD (CID2) and after 6-month of weight maintenance (CID3).

### Ethics

Local ethics committees approved the study, each patient provided written informed consent and the study was carried out in accordance with the principles of the Declaration of Helsinki. Committees included (1) Medical ethical commission from Maastricht University, NL (2) Copenhagen ethical research commission, DK (3) Bedfordshire local Research Ethics Committee, Luton and Dunstable Hospital NHS Trust, UK (4) Ethics Committee of the Faculty Hospital, Prague University, CZ (5) Ethical Commission by NMTI, Sofia, BG (6) Ethical Commission University Potsdam, D (7) Ethical Commission Medical University, Navarra, SP (8) Scientific council Heraklion general university hospital, Heraklion, GR and (9) Commission Cantonale d’ éthique de la recherche sur l’ être humain, Canton de Vaud, CH.

### Clinical variables

The following clinical variables were included in the analysis: body mass index (BMI), weight, body fat from bio-impedance and waist circumference. Several glycemic control measures were analyzed: fasting glucose/insulin; HOMA-IR (glucose (mmol/L) x insulin (mmol/L)/22.5); and Matsuda index, a measure of insulin-sensitivity derived from 2-hours oral glucose tolerance tests^19^. Total lipid levels (cholesterol, TAG, HDL and derived-LDL using the Friedwald formula) as well as total free fatty acid (FFA) were analyzed with blood biochemistry.

The Visceral Adiposity Index (VAI), a robust index of visceral fat, was derived using the formula proposed by Amato *et al*.^20^. This index is gender-specific and incorporates the following variables: BMI, waist circumference, total TAG and HDL-cholesterol. In this study, it was benchmarked against the Android-gynoid percent fat ratio as computed from dual-energy X-ray absorptiometry (DEXA) data available for 80 DiOGenes subjects from Denmark.

Additional indices pertaining to tissue-specific insulin-resistance (IR) were derived. This included the adipose-tissue IR (adipoIR^21^); the muscle insulin sensitivity and hepatic-IR indices^22^ (MISI and HIRI, respectively). AdipoIR corresponds plasma FFA x insulin levels. MISI is derived as the rate of decay of glucose concentration from its peak value to its nadir during Oral Glucose Tolerance Test (OGTT) divided by the mean insulin levels. HIRI is derived as the product of total area under curve (AUC) for glucose and insulin during the first 30 min of the (OGTT).

### Transcriptomics analysis

Abdominal subcutaneous adipose tissue (AT) biopsies were obtained by needle aspiration under local anesthesia after an overnight fast at baseline, at the end of LCD and at the end of weight maintenance. Biopsy samples were stored at −80 °C until total RNA extraction. Gene expression at baseline and upon LCD was quantified using 100-nt long paired-end RNA sequencing with an Illumina HiSeq 2000. This dataset has been previously described^9^ and data are available from the Gene Expression Omnibus under accession GSE95640.

High throughput quantitative real-time PCR (qPCR) was performed as previously described^9,23^ for specific lipogenesis genes (*FADS1/2*, *ELOVL5*, *FASN* and *SCD*) and leptin (*LEP*) and at all three intervention timepoints.

### Plasma omics analysis

Blood samples were taken after an overnight fasting period at baseline and upon completion of the LCD and WMD interventions. Figure 1C displays an overview of all omics analyses that were conducted in this study.

#### Lipidomics

LC-MS data generation has been described previously^16,24^. In total, 125 intact lipids including triacylglycerides, phosphocholines, sphingomyelins, cholesterol-esters, cholesterols and diacylglycerides were measured at all three intervention timepoints.

#### Metabolomics

Metabolomics data generation (^1^H NMR) was also described previously^24,25^ and quantified 18 low-weight metabolites related with obesity and insulin-sensitivity. Candidate mechanistic analyses focus on plasma ketone bodies (acetoacetate (AcAc) and beta hydroxy-isobutyrate (βHB)) quantified at all three intervention timepoints. Integrative analyses (predictive models) were pursued using all 18 metabolites.

#### Proteomics

Protein levels were measured using a multiplexed aptamer-based proteomic technology developed by SomaLogic Inc (Boulder, CO). This approach uses fluorescently labeled poly-nucleotide aptamers that recognize specific protein epitopes, similar to protein antibodies quantified using relative fluorescence on microarrays^26,27^. In total, 1,129 plasma proteins were measured, both at baseline and upon LCD. Proteomics data are available from the Open Science Framework at this URL https://osf.io/s4v8t/?view_only=90637f2941e14ec986e5888491fbdbbb.

### Quantification and statistical analysis

#### Lipid signature definition

The lipid signature was available from previous work^16^ and was computed using Principal Component Analyses (PCA) of the log_2_ fold-change for 79 lipids differentially expressed during LCD. The lipid signature was defined as Principal Component 1 (PC1). This continuous variable could be further dichotomized into responders (subjects with PC1 ≥ 0) and non-responders (subjects with PC1 < 0).

#### Association between lipid signature and clinical variables

Association with clinical variables was tested using linear mixed-effect models. The lipid signature (PC1), gender and age were modeled as fixed effects; center was modeled as a random effect. Analyses of a clinical endpoints at CID2 (LCD) or CID3 (weight maintenance) were adjusted for baseline levels at CID1. P-values were adjusted for multiple testing with the Benjamini-Hochberg procedure^28^. For visualization purpose, results are shown per group (responders/non-responders) and as time-series with the mean and its 95% confidence interval at a given CID. In these plots, ANOVA was used to compare differences between responders and non-responders at each CID; and adjust for gender, age and center. Comparisons between baseline parameters (BMI and Matsuda index) stratified by gender and responder/non-responder groups were made using ANOVA, followed with a Tukey Honest Significant Test for pairwise comparisons.

#### Pathway analyses

Enrichment analyses were performed using the KEGG^29,30^, REACTOME^31^ and PANTHER^32^ databases. Analyses were performed using a Fisher’s exact test. For Somalogic (proteomics) analyses, a background correction was implemented (by considering the universe as the 1129 proteins assayed on the array). Multiple testing adjustments were performed with resampling procedures (Monte-Carlo simulations). Significance levels were set at adjusted alpha 5%.

#### Gene expression analyses

Association between lipid signature and gene expression levels was tested as follows. For qPCR, linear mixed effect models were used (as for the analysis with clinical variables). For RNA-seq, due to the non-normality of the data, non-parametric approaches were used (ranksum test). Adjustment for multiple testing was performed using Benjamini-Hochberg correction.

All analyses were performed using R (v3.3.2)^33^.

#### Biochemical pathway analysis

*In-silico* biochemical pathway analyses were pursued to analyze connections between fatty acids involved in the TAG signature including (α-Linolenic Acid, Linoleic Acid, Stearic Acid, Palmitic Acid, Arachidonic Acid, Palmitoleic Acid and Oleic Acid) and Acetyl-CoA (as a key connection to ketometabolism). Objectives of this analysis were to identify the metabolic intermediates and the metabolic biotransformation routes potentially connecting the free fatty acids to ketometabolism. Methodologically, we used the KEGG database^34^ and specifically the PathComp methodology^35^ to identify the routes between the compounds of interest. We limited our search of paths to a maximum length of 10. Network analysis and visualization of paths and interconnectivities were performed using custom MATLAB (Mathwork Inc.) scripts and edited using yEd (yWorks GmbH).

#### Predictive models

Data were split into training and testing datasets. The training set included subjects from the two leading DiOGenes intervention centers (Denmark and the Netherlands) and the testing set included all remaining six centers. During weight maintenance, the two leading centers provided food freely to the participants^17,18^. We thus assumed that compliance to the randomized diet was higher for these two centers. Nevertheless, we also retrained models using a training set composed from a random subset over all eight centers.

Different types of predictive models were tested: Elastic Nets^36^, Random Forests^37^ and Gradient Boosting Machine(GBM)^38^. These models were fitted on the training set, using internal cross-validation (10-fold CV, repeated 5 times).

For elastic nets, model optimization was performed using the following grid of parameters: Alphas: 0 (ridge), 0.25, 0.50, 0.75, 1 (lasso) and lambdas: from 0.001 to 0.20, by equally spaced up to n = 40. For Random Forest, optimization was carried by testing different number of trees (up to 500). For GBM, optimization was carried over a grid with the following parameters: number of trees: 50, 500; max iteration depth 1.3; shrinkage 0.001, 0.005 and 0.01 and minimal terminal node size: 5, 10. The performance of a model was evaluated using Receiving Operating Curves (ROC curves). Models were tested using different set of features. The clinical model was based on 19 features (gender, weight, BMI, amount of fat mass, percentage of fat mass, waist circumference, VAI index, HOMA-IR, fasting glucose and insulin, Matsuda index, total cholesterol, HDL, LDL, triglycerides, fatty acids, adipose-IR, liver-IR and muscle-IR). Two sets of omics models were tested, the first set included previous clinical parameters and all features from lipidomics, metabolomics, and proteomics. This resulted in 1246 features. The second omics model, corresponded to a subset of the first model (with only 93 features), and was obtained by selecting features displaying marginal association with the responder status (Kruskal-Wallis analyses using only data from the training set, and with a very liberal significance level at alpha 30%) and also by discarding features that displayed either near-zero variance, were obtained through linear combinations from other features, or were highly correlated with another feature (Pearson R^2^ > 95%).

All analyses were performed using the caret R package^39^. Data were mean-centered and scaled to unit-variance by the caret preprocess function.

Evaluation of the ROC AUCs and their confidence intervals was performed using the pROC R package^40^. Comparison of any two ROC curves was made using the Delong’s test, as implemented in the roc.test function from the pROC package.

## Results

### Phenotyping and characterization of the responders and non-responders

Our study focuses on 375 overweight/obese, non-diabetic subjects from the DiOGenes cohort (with mean age 42 years old, and with 66% of women). These subjects followed first a weight loss intervention followed with a 6-month weight maintenance randomized intervention (Fig. 1A). By design, the weight maintenance intervention was only performed in subjects that had lost >8% of body weight.

Using a previously identify signature^16^ reflecting changes in plasma lipid composition during LCD, we could stratify subjects into two groups (responders or non-responders) with respect to glycemic improvements 6-month after LCD completion^16^ (Fig. 1B). This past study reported the lipid signature as a surrogate for both weight and glycemic improvements but did not aim to study the underlying physiology and molecular differences. In the present study, we perform a more comprehensive omics characterization of these two groups. Namely, we took advantages of other DiOGenes omics datasets, that encompassed transcriptomics, proteomics and metabolomics profiles (Fig. 1C). We also sought to study possible gender-specific differences and to pursue a deeper investigation from the lipid signature.

Previous analyses of clinical and physiological parameters had shown that these two groups (responders and non-responders) are comparable at baseline in term of weight and insulin sensitivity (Fig. 1D). Upon LCD, both groups had achieved significant weight loss (>8% initial body weight); yet the responder group had significantly higher weight loss (mean difference +2% improved weight loss with 95% CI (0.97, 2.34), two-sided t-test p = 2.58e-6). This same group also displayed significant improvements in insulin sensitivity (mean change in HOMA-IR index: −0.92 with 95% CI (−1.17, −0.66), p = 4.3e-11). The non-responders displayed no significant insulin sensitivity improvements (−0.25 with 95% CI (−0.64, 0.14), p = 0.21; Fig. 1D).

### Gender-specific characteristics

**Figure 2:**
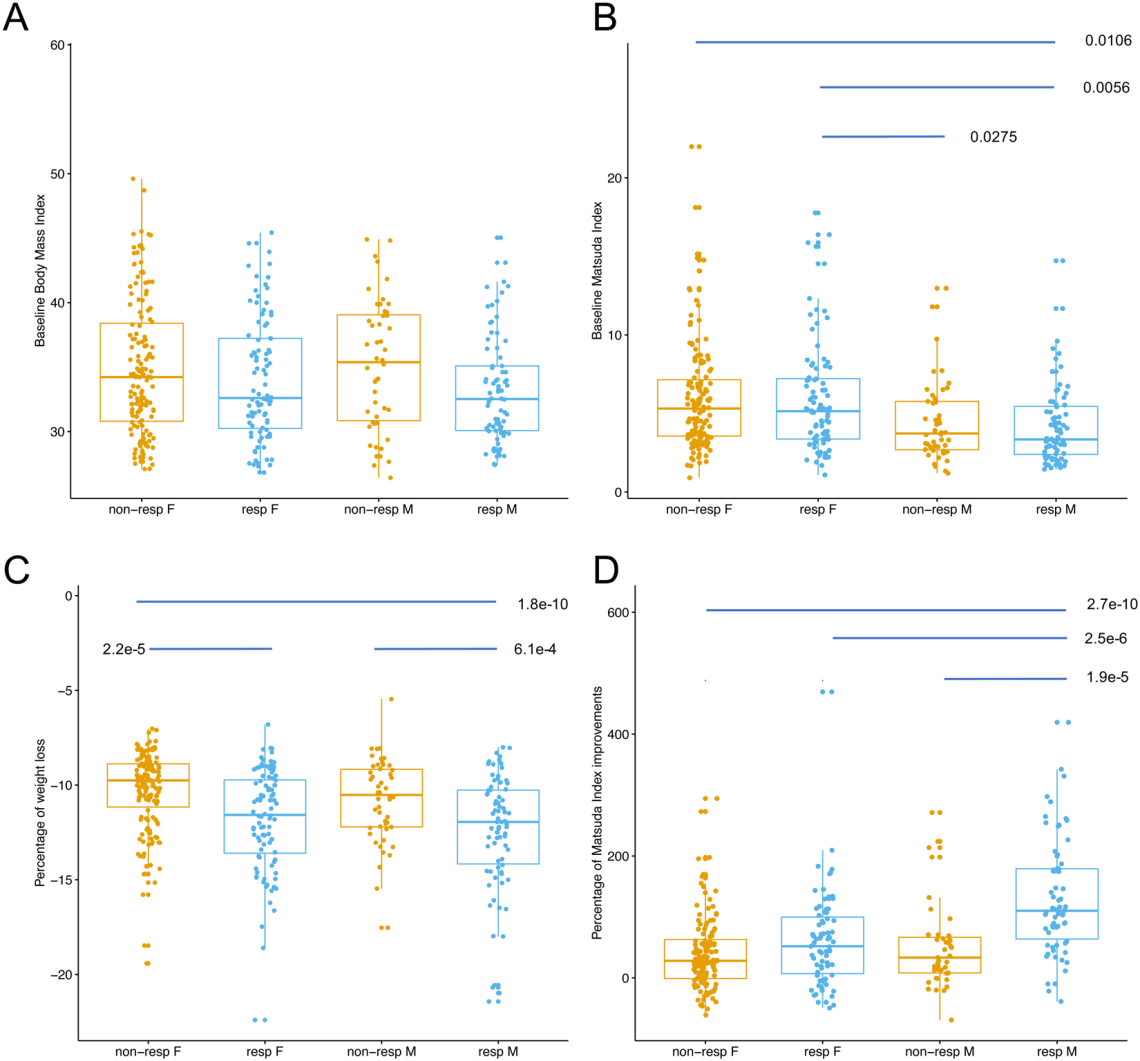
Boxplot of baseline BMI and Matsuda, and improvements during LCD. (**A**) Baseline BMI (**B**) baseline Matsuda index (**C**) percentage of BMI changes during LCD, relative to baseline (**D**) percentage of Matsuda index changes during LCD, relative to baseline. Groups are defined according to responder/non-responder status and gender (M/F). The displayed P-values were obtained from a Tukey Honest Significant Test (see Methods) and are adjusted for multiple testing. P-values greater than 5% are not shown.

We observed that 61% of the women were non-responders while this proportion was about 40% in men (odds ratio=2.48 with 95% CI (1.55, 3.90), Fisher’s exact test p = 5.3e-5). Pairwise comparisons between gender and responder status (Fig. 2 and Supplemental Table 1) showed that, independently of their responder status, men and women had similar baseline BMI, but that women were more insulin sensitive (higher Matsuda index) compared to men, suggesting less room for improvement during LCD. Indeed, upon LCD, responder men had significantly higher improvements of Matsuda index compared to their women counterpart (p = 2.5e-6, Fig. 2). With subsequent analysis, we sought to investigate the clinical and molecular differences between responders and non-responders, both globally (with gender-adjusted analyses) and in a gender-specific manner (gender-stratified analyses).

### Lipidomics profile characterization

The lipid signature is mostly composed of TAGs, which is consistent with results from total triglyceride levels showing that responders had significantly higher baseline levels than non-responders (p = 3.3e-6) and displayed significant down-regulation during LCD while non-responders did not display any significant changes during intervention (Supplemental Fig. 1). Consistent effects were found between men and women, with responders having higher baseline levels and stronger down-regulation during LCD and upon weight maintenance (Supplemental Table 2). Examination of individual lipids from the signature also confirmed these observations (Supplemental Fig. 2) and highlighted a shift in concentrations from TAGs of lower carbon number (42–54) and double bond (0–4).

### Characterization based on transcriptomics profile

To further investigate the role of adipose tissue in differences observed between responders and non-responders, we used RNAseq data for 350 genes previously documented as differentially expressed during LCD and linked with weight loss clinical outcomes^9^. Within this list, no gene displayed significant differences in baseline levels between responders and non-responders (smallest FDR = 0.44). At FDR < 5%, 15 genes had different evolution during LCD between responders and non-responders (Supplementary Table 3). Since such number of genes is not sufficient for pathway analyses, we relaxed the FDR cutoff to 10% and used 68 genes. Significant enrichments (FDR < 5%) were found in pathways pertaining to PPAR and leptin signaling; TAG and ketone body metabolism and fatty acid elongation (Supplementary Table 4). We observed that 15 genes (*ACADL, ACSL1, AKR1C2, ANGPT1, APBB1IP, CYP46A1, ELOVL5, GRIN2B, KLB, LEP, ME1, MECR, PTPLB, SCD*) were driving these enrichments; with *ELOVL5* and *SCD* involved in all fatty acid pathways.

Using targeted qPCR, we further investigated the expression of lipogenic genes including *FASN, SCD, FADS1, FADS2* and *ELOVL5* as well as *LEP* in adipose tissue biopsies. At baseline, both groups had comparable expression levels (nominal p > 5%, Supplemental Fig. 3). Following LCD, all these lipogenic genes distinguished responders from non-responders; with responders having significantly stronger down-regulation, including in gender-stratified analyses (FDR < 5%, Supplemental Table 5). The lipogenic genes all reached their initial levels during the weight maintenance phase. Leptin remained significantly down-regulated at study termination compared to baseline (p = 2.71e-6) and this down-regulation was significantly stronger in responders compared to non-responders (p = 0.0048).

### Characterization based on indices of visceral fat and tissue-specific insulin resistance

**Figure 3:**
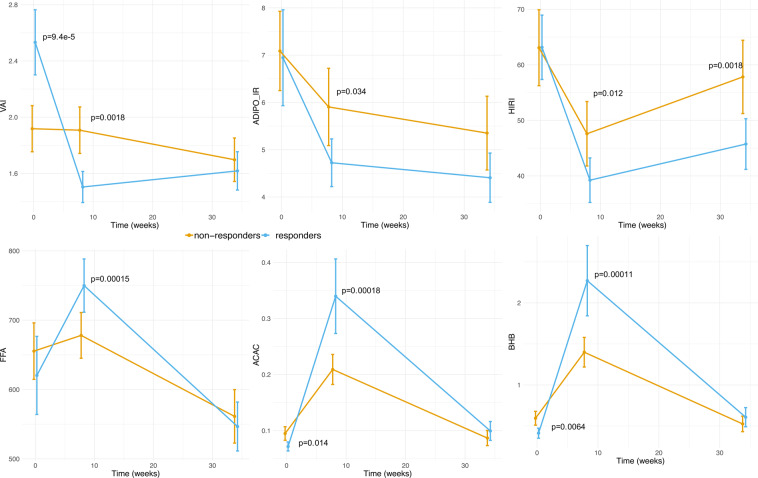
Evolution of visceral fat, tissue-specific IR indices, free fatty acid and ketone bodies during intervention. Data are shown as mean +/− 95% CI per group (responders/non-responders). P-values compare the difference between responders and non-responders at a given CID. Those p-values are adjusted for gender, age and center. P-values greater than 5% are not shown.

To further investigate the role and contribution of adipose tissue, we calculated visceral adiposity index (VAI) using BMI, waist circumference, total triglycerides and HDL as a surrogate marker of adipose tissue dysfunction as described previously^20^. While the groups were comparable, at baseline, in term of central obesity (as assessed with weight and BMI), they significantly differed in their Visceral Adiposity index (VAI), with responders having significantly higher baseline indices (p = 9.39e-5). Longitudinal analyses of the VAI showed that responders achieved significant reduction of visceral fat upon LCD and were able to maintain such reduction (Fig. 3). Conversely, non-responders did not show any significant changes in visceral fat content both upon LCD and weight maintenance. Gender-stratified analyses confirmed all these observations (Supplemental Table 2). Based on a subset of the DiOGenes cohort (N = 80 Danish subjects) and with ANOVA analyses adjusted for gender, we observed significant differences in DXA data (android-gynoid percent fat ratio at baseline) between responders and non-responders (p = 4.87e-7). Stratified analyses by gender reached the same conclusions (with p = 0.001 and 0.016 respectively for the male- and female-stratified analyses). Altogether these results show that responders had more visceral fat than non-responders, independently of gender.

Transcriptomics profiles, VAI and DXA indicated to the contribution of adipose tissue to the observed stratification and to glycemic outcome. Thus, we sought to further investigate the contribution of other organs by evaluating established indices of tissue-specific insulin resistance (adipose tissue, liver, and muscle; see Methods). At baseline, the two groups were comparable for all three indices (Supplemental Table 1), which is consistent with their overall insulin sensitivity (as assessed with fasting glucose/insulin levels; HOMA-IR and Matsuda index). Significant differences between groups were observed in changes in adipoIR index upon LCD (p = 0.008) and weight maintenance (p = 0.02). Specifically, responders displayed significantly better improvements than non-responders (Fig. 3). Significant differences were also observed in changes in hepatic insulin resistance (HIRI), both upon LCD (p = 0.002) and weight maintenance (p = 1.6e-4, Fig. 3) and were confirmed with gender-stratified analyses (Supplemental Table 2). No significant differences, independently of gender, were observed for improvements in muscle-specific IR (MISI), neither upon LCD or weight maintenance.

Finally, we compared plasma total levels of free fatty acids (FFA). No significant differences were observed at baseline. Overall, subjects significantly reduced their FFA levels at study termination (mean change = −83.32 mmol/L with 95% CI (−120.33, −46.30), p = 1.38e-5). During LCD, responders displayed significant higher increase in FFA than non-responders (p = 1.5e-4, see Fig. 3) but had comparable levels upon weight maintenance (p = 0.65). Gender-stratified analyses showed significant differences in women (p = 0.002) but no differences in men (p = 0.19). Since this study included twice more women than men, this may possibly due to a lack of statistical power.

### Alteration in ketone bodies

Metabolomics analysis revealed significant difference in level of plasma ketone bodies including AcAc and βHB between responders and non-responders at the baseline as well as during intervention; with consistent effects in males and females (Supplemental Table 2). Specifically, responders had significantly lower baseline levels and higher increase in ketone bodies during LCD, yielding to higher levels at LCD termination (Fig. 3). During weight maintenance, both groups displayed reduction in ketone bodies but at study termination both groups had comparable levels (p > 0.1).

### Biochemical connections between fatty acids and Acetyl-CoA

**Figure 4:**
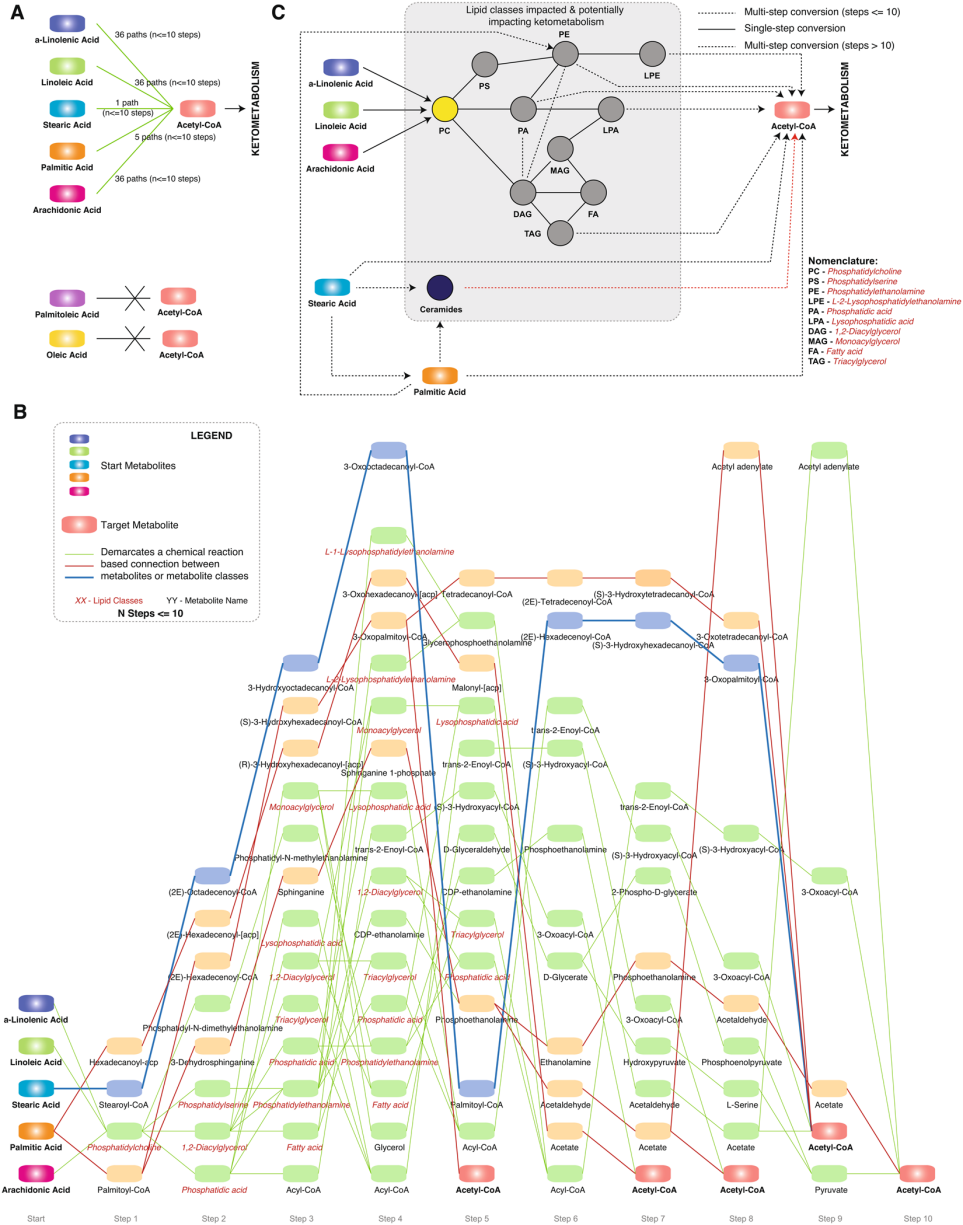
Biochemical pathway analysis for α-Linolenic Acid, Linoleic Acid, Stearic Acid, Palmitic Acid, Arachidonic Acid, Palmitoleic Acid and Oleic Acid to Acetyl-CoA. (**A**) Number of paths for connecting each compound to Acetyl-CoA. (**B**) Enumeration of the paths (steps <= 10). (**C**) Overview of the key components identified to play a key role in connecting the lipids to Acetyl-CoA.

Given the differences between the responders and the non-responders in terms of free fatty acids, TAGs and ketone bodies, we subsequently used biochemical pathway analysis (details in materials and methods) to identify the mechanistic linkages between fatty acids (α-Linolenic Acid, Linoleic Acid, Stearic Acid, Palmitic Acid, Arachidonic Acid, Palmitoleic Acid and Oleic Acid), that were enriched in triglycerides, and Acetyl-CoA (as a key component of ketometabolism). Using this approach, with a maximum route length of 10, we identified several routes connecting α-Linolenic Acid (36 routes), Linoleic Acid (36 routes), Stearic Acid (1 route), Palmitic Acid (5 routes) and Arachidonic Acid (36 routes) to Acetyl-CoA (Fig. 4A). However, no feasible mechanistic links were identified for connecting Palmitoleic Acid and Oleic Acid to Acetyl-CoA.

All the routes with the details of the intermediate metabolites for these connections are depicted in Fig. 4B. Results of this analysis indicate that the shortest route to impact ketometabolism via Acetyl-CoA is by Palmitic Acid (5 steps, *Palmitic acid - Palmitoyl-CoA - (2E)-Hexadecenoyl-CoA - (S)–3-Hydroxyhexadecenoyl-CoA – 3-Oxopalmitoyl-CoA – Acetyl-CoA*). Besides the direct route, Palmitic acid can also alter Acetyl-CoA levels via biochemical transformation routes involving Ceramides and Phosphatidylethanolamines (steps >10, Supplemental Fig. 4). Similarly, Stearic Acid can directly alter Acetyl-CoA levels (9 steps) and via routes involving Palmitic Acid and Ceramides. α-Linolenic Acid, Linoleic Acid and Arachidonic Acid were all predicted to impact Acetyl-CoA levels via Phosphatidylcholines. The predicted biochemical transformation routes involved lipid classes like Phosphatidylserine (PS), Phosphatidylethanolamine (PE), Lysophosphatidylethanolamine (LPE), Phosphatidic acid (PA), Lysophosphatidic acid (LPA), Monoacylglycerol (MAG), Diacylglycerol (DAG), Triacylglycerol (TAG) and Fatty acids (FA) (Fig. 4C). Based on biochemical transformations, TAG, LPA, PA, PE and LPE could all alter Acetyl-CoA levels and result in alteration in ketone bodies. The overall biotransformation space (Fig. 4C), connecting FFAs and Acetyl-CoA allows us to comprehend the aforementioned observations of significant increase in FFAs and ketometabolism in responders and reduction in FFAs as well as the accumulation of specific TAGs with lower carbon number (42–54) and double bond (0–4) in Non-Responders.

### Characterization based on proteomics revealed alteration in ApoE

**Figure 5:**
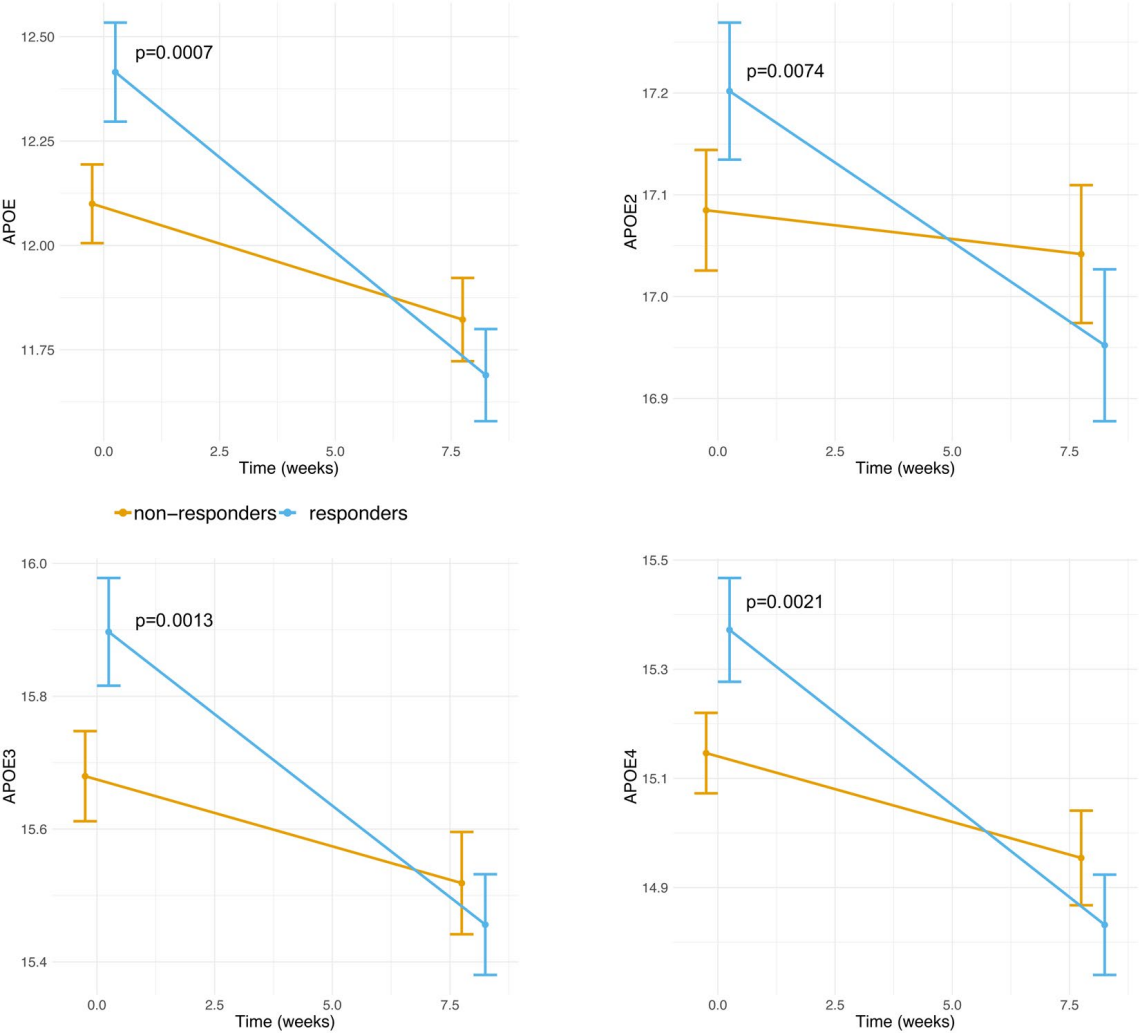
Evolution of somamers pertaining to ApoE during LCD. Data are shown as mean +/− 95% CI per group (responders/non-responders). P-values compare the difference between responders and non-responders at a given CID. Those p-values are adjusted for gender, age and center. P-values greater than 5% are not shown. Values on the Y axis correspond to relative fluorescence units, as provided by the Somalogic platform.

We took advantages of a large proteomics dataset (1,129 proteins) quantified from fasting plasma samples at baseline and after LCD^41^. We first assessed whether differences between the two groups could be observed in their baseline protein levels. Following adjustment for multiple-testing (FDR < 5%), four somamers (probes) had significant differences in baseline levels between responders and non-responders. All these somamers pertained to the ApoE protein (Supplemental Table 6). We observed that responders had higher baseline ApoE protein levels compared to non-responders (Fig. 5). During LCD, responders had significant down-regulation in ApoE levels while non-responders displayed no significant changes.

We evaluated changes in protein levels during LCD and whether these changes associated with the lipid signature. In total, 111 somamers (including the four ApoE somamers), pertaining to 106 unique proteins distinguished responders from non-responders (Supplemental Table 7). Similar APOE effects were seen in males and females (Supplemental Table 2), both for baseline levels and changes during LCD.

**Figure 6:**
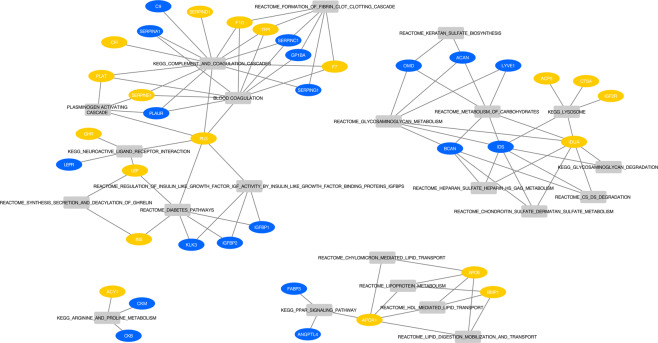
Pathways significantly enriched in proteins whose changes in levels during LCD distinguish responders from non-responders. This network displays the connections between the constituent proteins (shown as ellipses) and the enriched biological pathways (rectangles); the node color indicates whether protein levels at LCD termination (CID2) are more pronounced in non-responders (yellow edges) or responders (blue).

Subsequent pathway analyses of these 106 proteins showed significant enrichment in the 22 pathways definitions (Supplemental Table 8). A processed network summary is shown in Fig. 6 and highlights that these enrichments are confined to few specific biological processes: coagulation and complement factors, insulin signaling, glycosaminoglycan, lysosome, and lipoprotein metabolism.

### Integrative analyses to predict responder’s status using baseline information

Finally, we aimed to define predictive model, solely based on baseline parameters that would enable to predict the responder/non-responder status, prior to any clinical intervention. Whilst most clinical parameters did not display strong separation between the two groups, there were still a few such as triglyceride levels, gender, visceral adiposity index that showed significant differences; and additional parameters (e.g. cholesterol, BMI) with only marginal association (as presented previously). We hypothesized that perhaps the combination from all easily accessible clinical parameters may yield some level of prediction. And indeed, using models based on baseline anthropometric parameters, total lipid levels, insulin sensitivity measures (including tissue-specific), as well as gender and age,. Some modest performance was achieved (on testing set, ROC AUC = 61%, with 95% CI [51%, 71%]) However, this performance was not sufficient for prediction in a clinical setting (with the lower confidence interval very close to 50%, indicating close to random prediction). Analysis using different types of models (elastic nets, random forests and boosting) all yielded to similar performance, further confirming the difficulty to predict responders and non-responders solely based on clinical parameters.

**Figure 7:**
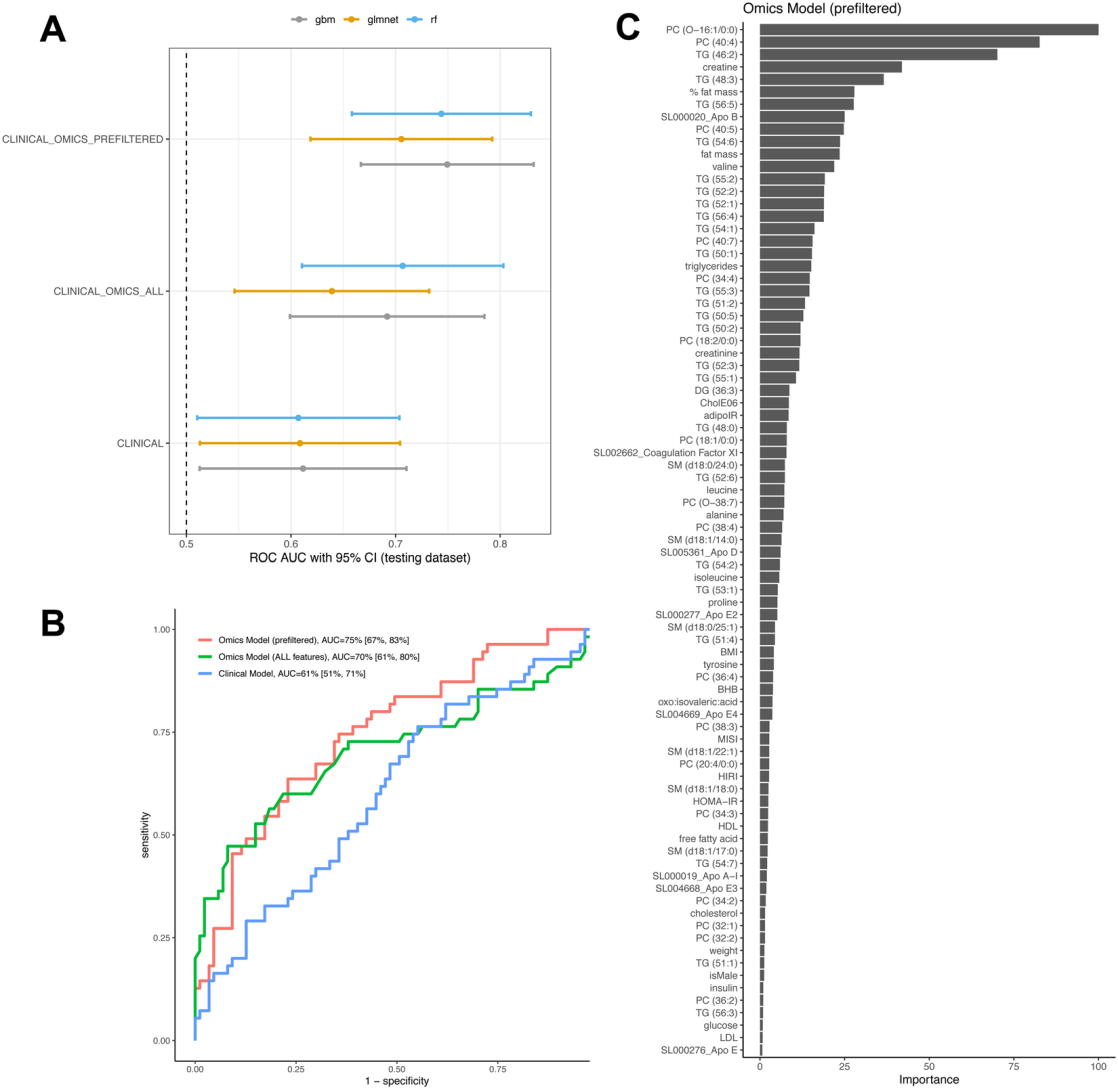
Predictive models based on baseline clinical and plasma omics. (**A**) Receiving Operating Curves (ROC) Area Under the Curve (AUC) for each model (3 set of features × 3 type of models). Performance is evaluated on the testing set (not used for constructing the models). AUCs and their 95% Confidence Intervals are shown. The line at AUC 50% indicates the performance from a random predictor. Models based on clinical parameters uses as input 19 features, the full omics model includes 1246 features, while the prefiltered omics model is a subset and uses 93 features as input (see Methods for full details). (**B**) ROC curves (on testing data) for the best models from the 3 set of features. The clinical model is based on an elastic net (glmnet), the Omics model with all features is a random forest, while the Omics models with prefiltered features is a gradient boosting machine. (**C**) Feature importance plot for the features retained by the gradient boosting machine model (constructed using the prefiltered Omics features as input).

In a second step, we aimed to construct predictive models using plasma omics parameters. Adipose tissue transcriptomics is not easily accessible in clinical routine, and thus was not considered in these analyses. This strategy yielded better models (see Fig. 7A,B), with the top omics model reaching AUC = 75% with 95% CI [67%, 83%], and outperformed significantly the clinical model (Delong’s two-sided pvalue = 0.00075). The top omics model is based on a gradient boosting machine, and incorporate 83 distinct features, whose relative importance is displayed in Fig. 7C. This plot reveals that the top features pertains mostly to TAGs, several indicators of fat mass, apolipoproteins (Apo E, Apo B, Apo D), indicators of insulin sensitivity (both systemic with HOMA-IR and peripheral with adipose IR, and lesser contribution from liver and muscle IR). Gender was also retained by the model, but only ranked as 77/83; indicating it had only small contribution to predicting the responder/non-responder status. Interestingly, tissue-specific indices of insulin resistance (adipose, liver and muscle) all ranked better (i.e. were more informative), than HOMA-IR or individual fasting or insulin levels. This highlights the importance of tissue-specific indicators for better diagnostic, as opposed to relying on general metabolic indicators.

Our models were trained using the two leading intervention centers (“shop-centers”) and validated with the remaining centers (“non-shop”). The rationale was that the shop-centers provided freely food during weight maintenance and thus compliance to the intended randomized diet was possibly higher. Re-analyses using a training set based on a random sampling across all centers, and validation with the remaining data yielded models with similar performance (Supplemental Fig. 7**)**. This further demonstrated the robustness of our models.

## Discussion

We previously stratified subjects according to their glycemic improvements (6-month after LCD completion) based on plasma lipid signature^16^. In this study we aimed to further characterize these two groups using combination of multi-omics and biochemical pathway modeling which allowed us to get more insights into the underlying mechanism and physiological changes that differentiate these two groups.

We observed that women were more likely non-responders than men, yet at baseline, women already displayed significantly better insulin sensitivity (both systemic sensitivity as assessed with Matsuda index and HOMA-IR, but also tissue-specific as estimated with proxies for the adipose, muscle and liver). Our main analyses were performed using gender as a covariate to maximize the statistical power^42^. In addition, we repeated our analyses using a gender-stratified approach. While the latter approach is considered suboptimal in term of statistical power, we could still confirm that the direction of effects for both clinical and omics key parameter was consistent between genders (Supplemental Figs. 5–6**)**. This suggests that the higher prevalence of non-responders with the women group, is more likely due to a better baseline condition rather than gender-specific response to LCD. Providing support to this hypothesis, in obesity studies and also clinical practice, enrolled women are relatively healthier than men in term of glycemic control. Within the PREVIEW study^43^, currently the largest weight loss study (N = 2,500); women have a significantly less pronounced baseline insulin resistance than men (mean HOMA-IR = 3.5 +/− 2.2 SD in women vs 4.24 +/− 2.82 in men; p < 0.001), and generally, a lesser Metabolic Syndrome score (mean Z-score 2.4 +/− 3.2 SD in women vs. 2.9 +/− 3.3 in men, p < 0.001). Also, in line with our observation that women display lesser insulin sensitivity improvements than men, the PREVIEW study found that men displayed better improvement of the Metabolic Syndrome status than women (p < 0.001). This yields important considerations for clinical practice. First, differences in metabolic improvements between gender may be due to a difference in baseline status; and might not be due, or only partially, to gender-specific difference in metabolism and physiology. Whilst this seems obvious, such differences are not discussed in the scientific literature and are ignored in weight loss management interventions. Future studies investigating gender-specific differences should pay attention to such baseline differences.

Interestingly in our study, we found that the directions of changes were consistent between genders, albeit smaller in women. This still prompt the needs elucidate the metabolic differences underlying biomarkers predicting responders and non-responders to weight loss intervention.

We observed that the identified lipid signature that distinguished responders from non-responders was due to a shift in fatty acid composition in TAG species. That indicates, not only increase in total level of TAGs but also alteration in fatty acid biosynthesis. Most of these TAGs have saturated monounsaturated fatty acyl chains, indicating *de novo* lipogenesis (DNL) as a main contributor to these specific changes^44^. This hypothesis is in agreement with studies demonstrating that the DNL pathway is stimulated in hyper-insulinemic obese subjects compared to normo-insulinemic obese subjects and associated with high blood TAGs concentrations, possibly exacerbating insulin resistance^45^.

While our study was limited with regards to access to liver or muscle biopsies, we were able to highlight tissue-specific effects between the two groups. Using both Visceral Adiposity Index and DXA data (android-gynoid ratio), we found that responders had higher visceral fat at baseline compared to non-responders. Further analyses during LCD and with adjustments for baseline visceral fat levels, show that visceral fat is significantly reduced only for responders (non-responders do not show any significant changes). These improvements in visceral fat are consistent with their significant improvement in insulin sensitivity in responders (compared to non-responders). And indeed, further investigation using established indices of tissue-specific insulin resistance (adipose tissue, liver, and muscle) confirmed baseline similarity between the two groups; and that responders had significant improvements in both adipose and hepatic insulin resistance indices (adipoIR and HIRI, respectively) upon LCD. These concomitant visceral fat reduction and glycemic improvements in responders is consistent with the expected physiological mechanisms^46^. It also emphasizes the need in clinical practice to assess both central adiposity (using BMI) and visceral fat (using indices such as VAI).

Gene expression analyses from subcutaneous adipose tissue biopsies provided additional evidence for difference in lipogenesis pathways (both with candidate qPCR approaches and using RNAseq data). Specifically, key lipogenic genes (*FASN, SCD, FADS1, FADS2* and *ELOVL5)*, as well as *LEP* provided consistent results with regards to their expected response to LCD and their role in improvements of insulin resistance^9,47–49^. These analyses further confirmed our hypothesis that alteration in lipid metabolism that differentiate responders from non-responders is, at least in part, due to the reduction in de novo lipogenesis in the visceral adipose tissue. Further investigation is required to elucidate the contribution of *de novo* lipogenesis in the liver to the alteration in lipid metabolism that differentiate responders from non-responders. However, from plasma proteomics, we observed a significant difference in the levels of total ApoE, ApoE2, ApoE3 and ApoE4 between responders and non-responders which is indicative of alteration in lipoprotein metabolism and shows a contribution of the liver to the observed alteration. ApoE is involved in many steps of lipoprotein homeostasis including triglyceride-rich VLDL and chylomicron remnants as well as subset of HDL particles^50^. Previous studies have shown that ApoE is not only important for fat accumulation, but is also linked with mechanisms of insulin resistance and the development of the metabolic syndrome^51–53^. ApoE has a wide tissue distribution and function^54^. While the liver is the major source of plasma ApoE, adipose tissue, including adipocytes and macrophages, is also producing a significant amount of this apoproteins^55^. Our proteomics pathway analyses highlighted other proteins involved in lipoprotein metabolism (APOA1, BMP1, FABP3 and ANGPTL4) and that are key markers in obesity and NAFLD^56–58^. Given the link between NAFLD, insulin resistance and lipid metabolism, these markers, as well as other adipo- and hepatokines would deserve further investigation in the context of weight loss and glycemic improvements following LCD intervention^59^.

Finally, since ketone bodies in mammals are produced predominantly in the liver from fatty acid oxidation and are important component of several important metabolic pathways such as β-oxidation (FAO), the tricarboxylic acid cycle (TCA), gluconeogenesis and de novo lipogenesis (DNL), we sought to assess if the circulating levels are differentiated between responders and non-responders. *In-silico* biochemical pathway analysis, leveraging known biochemical transformation routes, further substantiates the possibility that alterations in the identified lipid signature related to fatty acid partitioning in triglycerides could result in alterations in keto-metabolism and significant changes in circulating ketone bodies. This is also in agreement with changes that we observed with apolipoprotein that further confirms the potential contribution of liver in alteration in TAG-rich VLDL secretion.

The availability of both detailed baseline clinical parameters and plasma omics enabled us to construct and evaluate predictive models. These analyses demonstrated the need to acquire omics as a model solely based on clinical information was not able to achieve good performance (AUC = 61%, with 95% CI [51%, 71%]). By contrast, a more sophisticated model, based on 83 features increased significantly the performance (reaching AUC = 75%, with 95% CI [67%, 83%]). This model contrasts with our previous work^16^, whereby the classification into responders/non-responders was only possible using the lipid profile evolution during clinical intervention. In other previous studies^9,24^, we developed models applied to predicting improvements from a single clinical outcome (either weight loss or glycemic control); while here our work aims to distinguishes two groups that differ in multiple endpoints (weight loss, glycemic control, dyslipidemia) suggesting more complex metabolic improvements and thus a much better physiological improvements regarding obesity-related co-morbidities. Our new model also contrasts with a recent cross-sectional study^60^ aiming to predict baseline HOMA-IR, which pertains to a relatively easier task than predicting the response to an intervention^24^. While our proposed model may appear relatively complex for implementation in clinical practice, it remains accessible for translational research and only rely on plasma omics, without the need for intrusive biopsies or measurements. Additionally, the ranking of these baseline predictors, together with our mechanistic characterization of responders/non-responders during intervention, constitute a comprehensive knowledge source to better understand the underlying physiology of non-responders and responders. Notably, this further stresses the usefulness of tissue-specific indicators of insulin resistance, relative to more general indicator such as HOMA-IR or fasting glucose/insulin levels. This is fully consistent with previous work^61^ on three distinct cohorts demonstrating the relevance of such indices in the space of cardiometabolic diseases and would merit further investigation in complex diseases.

In conclusion, our study demonstrates the utility of multi-omics approaches for patient stratification and highlights specific biological processes. Our study provides insights into the role of adipose and liver tissues in the context of metabolic improvement following low-caloric meal replacement interventions. Altogether the specific omics signatures and gender-specific baseline differences are useful to clinicians to better understand inter-subject variability. Furthermore, we propose an integrative plasma omics model, that is able to identify non-responders both for weight loss and insulin sensitivity improvements prior to any intervention. We are investigating the lipoprotein metabolism in another clinical study to provide further insights into the role of liver *de novo lipogenesis* and mechanism of actions which would allow us to design a better nutritional intervention for the non-responders.

## Supplementary information

Supplementary Information.
