# Supplementary material for: Integrative phenotyping of glycemic responders upon clinical weight loss using multi-omics
Source: Sci Rep. 2020 Jun 8;10:9236. doi: 10.1038/s41598-020-65936-8 (PMC7280519; doi:10.1038/s41598-020-65936-8)
Supplement: Supplementary file 1 — Supplementary Information. [file 41598_2020_65936_MOESM1_ESM.docx]

Integrative phenotyping of glycemic responders upon clinical weight loss using multi-omics

**Authors:** Armand Valsesia ^1^, Anirikh Chakrabarti^1^, Jörg Hager ^1^, Dominique Langin ^2,3,4^, Wim H.M. Saris ^5^, Arne Astrup ^6^, Ellen E. Blaak^5^, Nathalie Viguerie^2^, Mojgan Masoodi^1,7^

**Affiliations:**

1. Nestlé Institute of Health Sciences, Lausanne, Switzerland
2. INSERM, UMR 1048, Institute of Metabolic and Cardiovascular Diseases, Toulouse, France
3. University of Toulouse, Paul Sabatier University, Toulouse, France
4. Toulouse University Hospitals, Laboratory of Clinical Biochemistry, Toulouse, France
5. Department of Human Biology, NUTRIM, School of Nutrition and Translational Research in Metabolism, Maastricht University Medical Centre + (MUMC+), Maastricht, The Netherlands
6. University of Copenhagen, Department of Nutrition, Exercise and Sports, Faculty of Science, Copenhagen, Denmark
7. Institute of Clinical Chemistry, Inselspital, Bern University Hospital, Bern, Switzerland

**Supplemental Figure 1 Evolution of total triglycerides during intervention**

Data are shown as mean +/- 95% CI per group (responders/non-responders).

**Supplemental Figure 2 Evolution of the lipid signature during intervention**

Data are shown for responders and non-responder; with each lipid shown according to its carbon length and saturation (number of double carbon bonds). Values are rescaled according to the whole population; with red (blue) indicating an average lipid concentration higher (lower) in a specific subject group relative to the whole population.

**Supplemental Figure 3 Evolution of key lipogenesis genes and leptin during intervention.**

Data are shown as mean +/- 95% CI per group (responders/non-responders). P-values compare the difference between responders and non-responders at a given CID. Those p-values are adjusted for gender, age and center. Values on the Y axis correspond to relative expression compared to the qPCR normalizing gene (*GSUB* gene).

Supplemental Figure 4 Biochemical routes between fatty acids and Acetyl-CoA (with maximal route length of 15)

Supplemental Figure 5 Regression coefficients for all variables (baseline levels), global and gender-stratified analyses

These graphs display the effect from the responder/non-responder status (regression coefficients with standard error) onto the indicated parameter in the plot title. These analyses are conducted with baseline biomarker levels.

Analyses were conducted using linear-mixed effect models, with adjustment for gender and age as fixed effects and center as a random effect. For the stratified analyses by gender, age was used a fixed effect (and center as a random effect). Coefficients with standard errors overlapping the 0 line are not significant. The key message from these plots is to show the consistency in effect size between males and females, as well as the same effect directionality.

Supplemental Figure 6 Regression coefficients for all variables (changes during LCD), global and gender-stratified analyses

These graphs display the effect from the responder/non-responder status (regression coefficients with standard error) onto the indicated parameter in the plot title. These analyses are conducted with changes in biomarker levels during weight loss.

Analyses were conducted using linear-mixed effect models, with adjustment for gender and age as fixed effects and center as a random effect. For the stratified analyses by gender, age was used a fixed effect (and center as a random effect). Coefficients with standard errors overlapping the 0 line are not significant. The key message from these plots is to show the consistency in effect size between males and females, as well as the same effect directionality.

Supplemental Figure 7 Predictive models based on baseline clinical and plasma omics

Receiving Operating Curves (ROC) Area Under the Curve (AUC) for each model (3 set of features x 3 type of models). Performance is evaluated on the testing set (not used for constructing the models). AUCs and their 95% Confidence Intervals are shown. The line at AUC 50% indicates the performance from a random predictor. Models based on clinical parameters uses as input 19 features, the full omics model includes 1246 features, while the prefiltered omics model is a subset and uses 93 features as input (see Methods for full details). Here, the training set was made from a random subset of participants from all 8 centers (n=110), and testing set was made from all remaining participants (n=142).

Supplemental Table 1 Baseline clinical characteristics

Number corresponds to mean value +/- standard deviation. Pairwise differences were tested using ANOVA with adjustment for age and center.

| **variable (baseline levels)** | **All subjects (n=375)** | **Non-responders (n=201)** | **Responders (n=174)** | **p-value** | **FDR** | **All males (n=128)** | **Non-responders (n=50 males)** | **Responders (n=78 males)** | **p-value (males NR vs R)** | **FDR (males NR vs R)** | **All females (n=247)** | **Non-responders (n=151 females)** | **Responders (n=96 females)** | **p-value (females NR vs R)** | **FDR (females NR vs R)** | **p-value (NonResp females vs males)** | **FDR (NonResp females vs males)** | **p-value (Resp females vs males)** | **FDR (Resp females vs males)** |
| --- | --- | --- | --- | --- | --- | --- | --- | --- | --- | --- | --- | --- | --- | --- | --- | --- | --- | --- | --- |
| age, y | 42.15 +/- 6.36 | 41.70 +/- 6.30 | 42.68 +/- 6.41 | 0.4312 | 0.6903 | 43.16 +/- 6.04 | 41.96 +/- 5.95 | 43.94 +/- 6.01 | 0.05565 | 0.2666 | 41.63 +/- 6.47 | 41.62 +/- 6.42 | 41.66 +/- 6.58 | 0.8821 | 0.915 | 0.9974 | 0.9974 | 0.01529 | 0.02691 |
| weight, kg | 100.29 +/- 17.73 | 100.41 +/- 18.46 | 100.15 +/- 16.89 | 0.2915 | 0.5883 | 109.11 +/- 18.17 | 112.25 +/- 19.57 | 107.09 +/- 17.05 | 0.534 | 0.8314 | 95.73 +/- 15.68 | 96.50 +/- 16.35 | 94.51 +/- 14.56 | 0.6494 | 0.8632 | 2.42E-09 | 2.18E-08 | 3.89E-07 | 3.50E-06 |
| BMI, kg/m2 | 34.35 +/- 4.94 | 34.95 +/- 5.19 | 33.67 +/- 4.54 | 0.09314 | 0.4191 | 33.97 +/- 4.60 | 35.20 +/- 5.01 | 33.18 +/- 4.16 | 0.1042 | 0.2679 | 34.55 +/- 5.10 | 34.87 +/- 5.27 | 34.06 +/- 4.81 | 0.4595 | 0.8632 | 0.4244 | 0.5456 | 0.2662 | 0.2995 |
| Visceral Adiposity Index (VAI) | 2.20 +/- 1.37 | 1.92 +/- 1.16 | 2.53 +/- 1.52 | 9.39E-05 | 0.0008454 | 2.40 +/- 1.45 | 1.92 +/- 0.95 | 2.71 +/- 1.62 | 0.01553 | 0.1398 | 2.10 +/- 1.32 | 1.92 +/- 1.23 | 2.39 +/- 1.42 | 0.003585 | 0.03226 | 0.6684 | 0.8021 | 0.5838 | 0.5838 |
| body fat mass, % | 40.08 +/- 8.14 | 41.27 +/- 7.64 | 38.64 +/- 8.52 | 0.1447 | 0.521 | 32.44 +/- 7.23 | 33.80 +/- 8.31 | 31.42 +/- 6.18 | 0.06914 | 0.2666 | 43.99 +/- 5.33 | 43.96 +/- 5.24 | 44.05 +/- 5.48 | 0.6703 | 0.8632 | 5.65E-17 | 1.02E-15 | 3.44E-26 | 6.19E-25 |
| waist circumference, cm | 107.33 +/- 12.83 | 107.25 +/- 14.08 | 107.43 +/- 11.30 | 0.1804 | 0.5412 | 113.12 +/- 12.15 | 116.17 +/- 13.42 | 111.24 +/- 10.96 | 0.09606 | 0.2679 | 104.32 +/- 12.14 | 104.34 +/- 13.06 | 104.29 +/- 10.63 | 0.915 | 0.915 | 1.70E-08 | 1.02E-07 | 0.00024 | 0.00108 |
| glucose levels, mmol/L | 5.04 +/- 0.61 | 5.00 +/- 0.60 | 5.08 +/- 0.62 | 0.6992 | 0.7866 | 5.20 +/- 0.56 | 5.19 +/- 0.51 | 5.21 +/- 0.59 | 0.566 | 0.8314 | 4.96 +/- 0.62 | 4.94 +/- 0.61 | 4.98 +/- 0.62 | 0.8122 | 0.915 | 0.02309 | 0.03464 | 0.01645 | 0.02691 |
| insulin levels, SI units | 10.83 +/- 6.40 | 10.58 +/- 6.81 | 11.12 +/- 5.90 | 0.6064 | 0.7676 | 12.89 +/- 6.91 | 12.96 +/- 7.91 | 12.84 +/- 6.25 | 0.9382 | 0.9854 | 9.73 +/- 5.83 | 9.76 +/- 6.21 | 9.69 +/- 5.21 | 0.4343 | 0.8632 | 0.002843 | 0.007311 | 0.00103 | 0.003172 |
| HOMA-IR | 2.87 +/- 1.79 | 2.78 +/- 1.82 | 2.98 +/- 1.74 | 0.8168 | 0.8168 | 3.50 +/- 1.98 | 3.53 +/- 2.21 | 3.48 +/- 1.84 | 0.9854 | 0.9854 | 2.53 +/- 1.57 | 2.52 +/- 1.59 | 2.56 +/- 1.55 | 0.6714 | 0.8632 | 0.0005691 | 0.002049 | 0.001398 | 0.003594 |
| Matsuda index | 5.38 +/- 3.27 | 5.57 +/- 3.29 | 5.16 +/- 3.24 | 0.6396 | 0.7676 | 4.32 +/- 2.51 | 4.45 +/- 2.52 | 4.24 +/- 2.52 | 0.8441 | 0.9854 | 5.96 +/- 3.48 | 5.96 +/- 3.44 | 5.96 +/- 3.57 | 0.435 | 0.8632 | 0.01088 | 0.02176 | 0.001057 | 0.003172 |
| adiposity IR index | 7.02 +/- 5.66 | 7.09 +/- 5.18 | 6.95 +/- 6.14 | 0.8138 | 0.8168 | 7.27 +/- 5.85 | 7.14 +/- 4.86 | 7.35 +/- 6.43 | 0.6622 | 0.8513 | 6.83 +/- 5.52 | 7.07 +/- 5.35 | 6.47 +/- 5.78 | 0.4985 | 0.8632 | 0.834 | 0.8831 | 0.4618 | 0.489 |
| HIRI index | 63.12 +/- 42.22 | 63.08 +/- 46.36 | 63.16 +/- 37.23 | 0.3847 | 0.6903 | 74.73 +/- 46.96 | 80.75 +/- 56.93 | 71.16 +/- 39.90 | 0.6004 | 0.8314 | 56.70 +/- 37.97 | 57.14 +/- 40.77 | 56.01 +/- 33.30 | 0.6017 | 0.8632 | 0.003587 | 0.00807 | 0.0109 | 0.02179 |
| MISI index | 0.06 +/- 0.05 | 0.06 +/- 0.05 | 0.06 +/- 0.05 | 0.2638 | 0.5883 | 0.06 +/- 0.05 | 0.06 +/- 0.04 | 0.06 +/- 0.06 | 0.885 | 0.9854 | 0.06 +/- 0.05 | 0.06 +/- 0.05 | 0.05 +/- 0.04 | 0.1946 | 0.8632 | 0.7166 | 0.8062 | 0.1366 | 0.164 |
| total cholesterol, mmol/L | 4.84 +/- 1.03 | 4.74 +/- 0.99 | 4.96 +/- 1.07 | 0.02654 | 0.1593 | 5.02 +/- 1.12 | 4.88 +/- 1.18 | 5.11 +/- 1.08 | 0.07404 | 0.2666 | 4.75 +/- 0.97 | 4.69 +/- 0.91 | 4.83 +/- 1.05 | 0.1203 | 0.7221 | 0.2254 | 0.3121 | 0.106 | 0.1363 |
| LDL, mmol/L | 3.01 +/- 0.89 | 2.94 +/- 0.86 | 3.09 +/- 0.93 | 0.2942 | 0.5883 | 3.24 +/- 0.99 | 3.18 +/- 1.01 | 3.28 +/- 0.98 | 0.3087 | 0.6946 | 2.89 +/- 0.81 | 2.86 +/- 0.79 | 2.93 +/- 0.85 | 0.4978 | 0.8632 | 0.02267 | 0.03464 | 0.02048 | 0.03072 |
| HDL, mmol/L | 1.20 +/- 0.33 | 1.24 +/- 0.32 | 1.15 +/- 0.34 | 0.4602 | 0.6903 | 1.05 +/- 0.28 | 1.09 +/- 0.25 | 1.03 +/- 0.30 | 0.5286 | 0.8314 | 1.28 +/- 0.33 | 1.30 +/- 0.32 | 1.26 +/- 0.34 | 0.6034 | 0.8632 | 3.59E-06 | 1.61E-05 | 7.56E-06 | 4.54E-05 |
| triglyceride, mmol/L | 1.38 +/- 0.64 | 1.22 +/- 0.55 | 1.57 +/- 0.70 | 3.33E-06 | 5.99E-05 | 1.63 +/- 0.72 | 1.37 +/- 0.58 | 1.80 +/- 0.76 | 0.002624 | 0.04723 | 1.26 +/- 0.56 | 1.18 +/- 0.53 | 1.39 +/- 0.59 | 0.000545 | 0.00981 | 0.01275 | 0.02294 | 0.002568 | 0.005777 |
| free fatty acid levels, mmol/L | 638.51 +/- 302.73 | 655.39 +/- 258.28 | 620.36 +/- 344.19 | 0.6156 | 0.7676 | 559.86 +/- 290.35 | 552.45 +/- 176.16 | 564.61 +/- 345.32 | 0.5532 | 0.8314 | 696.04 +/- 299.41 | 703.49 +/- 276.58 | 684.32 +/- 334.02 | 0.8888 | 0.915 | 0.001043 | 0.003128 | 0.04147 | 0.05742 |

Supplemental Table 2 Results from Linear Mixed Effect models

Analyses were performed using linear mixed effect using the continuous signature (PC1) with adjustment for age and gender as fixed effects and center as a random effect. In gender-stratified analyses, gender was evidently not included as a covariable. Highlighted p-values in bold correspond to FDR < 5%.

|  | **Baseline levels** | | | | | | **Changes during LCD relative to baseline** | | | | |  | **Changes at study termination relative to baseline** | | | | | |
| --- | --- | --- | --- | --- | --- | --- | --- | --- | --- | --- | --- | --- | --- | --- | --- | --- | --- | --- |
| **parameter** | **Any Gender regression coefficients +/ SE** | **Any Gender P** | **Males regression coefficients +/ SE** | **Males P** | **Females regression coefficients +/ SE** | **Females P** | **Any Gender regression coefficients +/ SE** | **Any Gender P** | **Males regression coefficients +/ SE** | **Males P** | **Females regression coefficients +/ SE** | **parameter** | **Any Gender regression coefficients +/ SE** | **Any Gender P** | **Males regression coefficients +/ SE** | **Males P** | **Females regression coefficients +/ SE** | **Females P** |
| ACAC | -0.24 +/- 0.08 | **0.001744** | -0.14 +/- 0.08 | 0.06881 | -0.34 +/- 0.12 | 0.004742 | 1.89 +/- 0.35 | **9.75E-08** | 1.62 +/- 0.56 | **0.004638** | 1.93 +/- 0.44 | ACAC | 0.16 +/- 0.11 | 0.1604 | -0.02 +/- 0.20 | 0.9301 | 0.29 +/- 0.13 | 0.02839 |
| ADIPO_IR | 2.13 +/- 6.58 | 0.7469 | 7.19 +/- 9.27 | 0.4396 | -1.76 +/- 9.32 | 0.8501 | -11.52 +/- 4.33 | **0.008275** | -16.36 +/- 5.75 | **0.005363** | -5.26 +/- 6.22 | ADIPO_IR | -9.73 +/- 4.35 | 0.02626 | -6.68 +/- 6.50 | 0.3063 | -10.00 +/- 5.69 | 0.08124 |
| APOE | 3.56 +/- 0.68 | **3.10E-07** | 3.70 +/- 1.00 | **0.0003951** | 3.75 +/- 0.92 | **7.79E-05** | -3.19 +/- 0.54 | **9.49E-09** | -4.06 +/- 0.86 | **1.02E-05** | -2.63 +/- 0.69 | APOE | not measured at study termination | | | | | |
| APOE2 | 1.70 +/- 0.41 | **5.10E-05** | 1.90 +/- 0.62 | 0.002768 | 1.66 +/- 0.56 | 0.003521 | -1.43 +/- 0.33 | **2.60E-05** | -1.59 +/- 0.57 | 0.006682 | -1.31 +/- 0.42 | APOE2 | not measured at study termination | | | | | |
| APOE3 | 2.48 +/- 0.47 | **3.64E-07** | 2.71 +/- 0.69 | **0.0001765** | 2.53 +/- 0.65 | **0.000151** | -2.14 +/- 0.40 | **2.38E-07** | -2.66 +/- 0.65 | **0.0001036** | -1.85 +/- 0.52 | APOE3 | not measured at study termination | | | | | |
| APOE4 | 2.69 +/- 0.53 | **7.44E-07** | 2.90 +/- 0.78 | **0.0003508** | 2.73 +/- 0.72 | **0.00023** | -2.54 +/- 0.43 | **8.59E-09** | -3.12 +/- 0.67 | **1.18E-05** | -2.22 +/- 0.56 | APOE4 | not measured at study termination | | | | | |
| BHB | -1.72 +/- 0.55 | **0.001764** | -1.01 +/- 0.57 | 0.07847 | -2.34 +/- 0.83 | **0.005109** | 12.63 +/- 2.25 | **3.84E-08** | 10.94 +/- 3.67 | **0.003483** | 12.91 +/- 2.83 | BHB | 1.11 +/- 0.77 | 0.151 | -0.38 +/- 1.36 | 0.7811 | 2.20 +/- 0.91 | 0.01623 |
| BMI | -2.04 +/- 5.13 | 0.6906 | -5.82 +/- 7.04 | 0.4101 | 1.53 +/- 7.18 | 0.8319 | -5.87 +/- 0.90 | **2.50E-10** | -6.64 +/- 1.45 | **1.16E-05** | -5.46 +/- 1.18 | BMI | -8.27 +/- 2.11 | **0.0001036** | -8.18 +/- 2.86 | 0.005118 | -8.76 +/- 3.01 | 0.003907 |
| CHOL | 2.61 +/- 1.00 | **0.009236** | 3.34 +/- 1.72 | 0.0549 | 2.22 +/- 1.25 | 0.07571 | -4.57 +/- 0.65 | **1.15E-11** | -4.48 +/- 0.96 | **8.37E-06** | -4.62 +/- 0.88 | CHOL | -1.26 +/- 0.71 | 0.07418 | -0.39 +/- 1.17 | 0.739 | -1.99 +/- 0.89 | 0.02594 |
| ELOVL5 | -3.08 +/- 2.61 | 0.2389 | -2.44 +/- 2.49 | 0.3302 | -3.81 +/- 4.13 | 0.358 | -7.36 +/- 1.34 | **1.03E-07** | -7.52 +/- 1.87 | **0.00014** | -7.05 +/- 1.90 | ELOVL5 | -3.81 +/- 2.39 | 0.1117 | -2.00 +/- 2.66 | 0.4555 | -6.02 +/- 3.90 | 0.1253 |
| FADS1 | -0.73 +/- 1.06 | 0.4889 | -0.01 +/- 1.75 | 0.9955 | -1.30 +/- 1.35 | 0.3383 | -1.10 +/- 0.27 | **5.42E-05** | -1.67 +/- 0.55 | **0.003132** | -0.44 +/- 0.26 | FADS1 | -0.29 +/- 1.06 | 0.7859 | 0.24 +/- 1.78 | 0.8913 | -0.92 +/- 1.34 | 0.4935 |
| FADS2 | 0.09 +/- 0.91 | 0.9196 | 0.12 +/- 1.15 | 0.9187 | 0.30 +/- 1.34 | 0.8262 | -1.25 +/- 0.24 | **4.56E-07** | -1.40 +/- 0.45 | **0.002547** | -1.03 +/- 0.28 | FADS2 | -1.22 +/- 0.79 | 0.1222 | -0.34 +/- 0.92 | 0.7094 | -2.29 +/- 1.26 | 0.07039 |
| FASN | -2.17 +/- 1.22 | 0.07596 | -0.92 +/- 0.98 | 0.3487 | -3.44 +/- 1.96 | 0.08152 | -2.12 +/- 0.42 | **1.13E-06** | -1.69 +/- 0.55 | **0.002861** | -2.53 +/- 0.61 | FASN | -0.97 +/- 0.95 | 0.3115 | -0.34 +/- 1.08 | 0.7581 | -1.56 +/- 1.53 | 0.311 |
| FAT_PERC | -6.82 +/- 6.72 | 0.3106 | -15.12 +/- 12.49 | 0.2287 | -0.69 +/- 7.70 | 0.9285 | -8.02 +/- 4.97 | 0.108 | -15.11 +/- 8.31 | 0.07241 | -5.72 +/- 6.05 | FAT_PERC | -13.75 +/- 5.22 | 0.008925 | -9.18 +/- 7.46 | 0.2216 | -15.94 +/- 7.29 | 0.03011 |
| FFA | 9.38 +/- 328.63 | 0.9772 | -167.20 +/- 433.23 | 0.7002 | 138.29 +/- 496.68 | 0.781 | 751.87 +/- 239.91 | **0.001902** | 403.94 +/- 308.01 | 0.1923 | 1149.09 +/- 367.23 | FFA | 134.70 +/- 246.73 | 0.5855 | -42.20 +/- 339.03 | 0.9012 | 370.84 +/- 362.84 | 0.3084 |
| GLU | 0.77 +/- 0.61 | 0.2067 | 1.04 +/- 0.87 | 0.237 | 0.75 +/- 0.84 | 0.3757 | -1.63 +/- 0.41 | **8.98E-05** | -2.97 +/- 0.60 | **3.22E-06** | -0.46 +/- 0.55 | GLU | -0.77 +/- 0.49 | 0.1165 | -0.59 +/- 0.78 | 0.4472 | -0.74 +/- 0.62 | 0.2358 |
| HDL | -0.51 +/- 0.31 | 0.1013 | -1.00 +/- 0.39 | 0.01073 | -0.19 +/- 0.45 | 0.6777 | 0.46 +/- 0.20 | 0.02438 | 0.47 +/- 0.31 | 0.1332 | 0.48 +/- 0.27 | HDL | 0.68 +/- 0.23 | 0.00366 | 0.74 +/- 0.33 | 0.02674 | 0.82 +/- 0.32 | 0.01181 |
| HIRI | -16.32 +/- 45.49 | 0.72 | -60.06 +/- 78.05 | 0.4432 | 16.80 +/- 56.12 | 0.765 | -109.67 +/- 35.22 | **0.002048** | -166.07 +/- 71.63 | 0.02285 | -61.30 +/- 36.97 | HIRI | -146.24 +/- 38.28 | **0.0001637** | -148.81 +/- 67.92 | 0.03084 | -124.59 +/- 43.79 | 0.00496 |
| HOMA_IR | 2.74 +/- 1.85 | 0.1383 | 5.27 +/- 3.14 | 0.0958 | 0.46 +/- 2.28 | 0.8404 | -8.28 +/- 1.82 | **7.87E-06** | -14.32 +/- 4.22 | **0.0009892** | -4.03 +/- 1.32 | HOMA_IR | -6.71 +/- 2.29 | 0.003639 | -7.32 +/- 3.62 | 0.04565 | -5.44 +/- 2.95 | 0.06682 |
| INS | 7.52 +/- 6.59 | 0.255 | 17.10 +/- 10.89 | 0.1189 | -0.93 +/- 8.26 | 0.9105 | -25.98 +/- 5.67 | **6.56E-06** | -44.97 +/- 12.96 | **0.0007595** | -13.01 +/- 4.56 | INS | -25.23 +/- 7.87 | **0.001478** | -28.78 +/- 14.82 | 0.05467 | -19.08 +/- 8.68 | 0.02908 |
| LDL | 1.31 +/- 0.89 | 0.1394 | 2.03 +/- 1.54 | 0.1918 | 0.94 +/- 1.09 | 0.3892 | -2.47 +/- 0.58 | **2.40E-05** | -2.03 +/- 0.93 | 0.0301 | -2.70 +/- 0.76 | LDL | -0.51 +/- 0.60 | 0.3922 | 0.08 +/- 0.95 | 0.9362 | -1.22 +/- 0.78 | 0.1161 |
| LEP | 12.87 +/- 9.87 | 0.1932 | 2.74 +/- 10.85 | 0.8016 | 22.63 +/- 15.23 | 0.1394 | -24.27 +/- 6.32 | **0.0001571** | -22.27 +/- 5.93 | **0.0003552** | -26.91 +/- 10.13 | LEP | -28.96 +/- 10.15 | 0.004797 | -7.04 +/- 9.25 | 0.4494 | -57.48 +/- 17.16 | **0.001073** |
| MATSUDA | -2.50 +/- 3.39 | 0.4607 | -5.27 +/- 4.05 | 0.1964 | -0.48 +/- 5.01 | 0.9244 | 12.63 +/- 3.61 | **0.000538** | 23.74 +/- 5.81 | **9.06E-05** | 2.90 +/- 4.59 | MATSUDA | 8.49 +/- 3.39 | 0.01284 | 11.16 +/- 5.00 | 0.02765 | 5.56 +/- 4.63 | 0.2308 |
| MISI | -0.07 +/- 0.06 | 0.2164 | 0.01 +/- 0.09 | 0.9013 | -0.12 +/- 0.07 | 0.0984 | 0.06 +/- 0.06 | 0.2688 | 0.17 +/- 0.09 | 0.05742 | -0.06 +/- 0.07 | MISI | -0.03 +/- 0.06 | 0.5431 | 0.03 +/- 0.10 | 0.7374 | -0.10 +/- 0.07 | 0.1696 |
| SCD | -1.70 +/- 19.45 | 0.9305 | 17.83 +/- 18.09 | 0.3278 | -18.00 +/- 30.85 | 0.5606 | -32.14 +/- 8.22 | **0.0001225** | -17.79 +/- 12.34 | 0.1537 | -45.69 +/- 11.21 | SCD | -37.72 +/- 17.93 | 0.03674 | -22.79 +/- 19.38 | 0.2445 | -57.46 +/- 29.09 | 0.0505 |
| TRIG | 3.95 +/- 0.64 | **1.48E-09** | 5.18 +/- 1.11 | **7.93E-06** | 2.97 +/- 0.78 | **0.0001725** | -5.23 +/- 0.36 | **9.09E-38** | -6.08 +/- 0.58 | **2.48E-18** | -4.60 +/- 0.45 | TRIG | -2.75 +/- 0.49 | **3.02E-08** | -1.87 +/- 0.91 | 0.04348 | -3.23 +/- 0.56 | **2.82E-08** |
| VAI | 7.69 +/- 1.41 | **9.39E-08** | 10.38 +/- 2.19 | **6.41E-06** | 5.56 +/- 1.85 | 0.002907 | -9.80 +/- 0.77 | **1.82E-30** | -9.64 +/- 1.16 | **2.76E-13** | -9.08 +/- 0.97 | VAI | -5.23 +/- 0.84 | **1.25E-09** | -3.26 +/- 1.34 | 0.0165 | -6.48 +/- 1.07 | **6.90E-09** |
| WAIST | -0.80 +/- 12.85 | 0.9503 | -17.62 +/- 19.07 | 0.3573 | 16.57 +/- 17.25 | 0.3377 | -13.13 +/- 4.75 | **0.006004** | -19.62 +/- 6.50 | **0.003159** | -9.71 +/- 6.65 | WAIST | -13.04 +/- 7.56 | 0.08551 | -14.45 +/- 10.52 | 0.1725 | -14.99 +/- 10.61 | 0.1593 |
| WEIGHT | -5.68 +/- 17.15 | 0.7408 | -14.83 +/- 27.42 | 0.5897 | 4.83 +/- 22.02 | 0.8267 | -17.14 +/- 2.64 | **2.82E-10** | -20.29 +/- 4.65 | **2.75E-05** | -15.18 +/- 3.21 | WEIGHT | -23.45 +/- 6.13 | **0.0001529** | -24.81 +/- 9.10 | 0.007398 | -23.36 +/- 8.42 | 0.005981 |

Supplemental Table 3 Results from RNAseq analyses

| **ENSEMBL GENE ID** | **HGNC SYMBOL** | **Chromosome** | **Start** | **End** | **Strand** | **Baseline Mean expression (FPKM)** | **Log2 Fold Change** | **pvalue** | **FDR** |
| --- | --- | --- | --- | --- | --- | --- | --- | --- | --- |
| ENSG00000176273 | SLC35G1 | 10 | 95653730 | 95715819 | 1 | 275.98 | 0.25 | 0.0001 | **0.0301** |
| ENSG00000260578 | ENSG00000260578 | 18 | 65149028 | 65152203 | -1 | 113.68 | -0.20 | 0.0002 | **0.0301** |
| ENSG00000206538 | VGLL3 | 3 | 86987119 | 87040269 | -1 | 2533.94 | -0.17 | 0.0005 | **0.0367** |
| ENSG00000126016 | AMOT | X | 112017731 | 112084043 | -1 | 1000.29 | -0.24 | 0.0007 | **0.0367** |
| ENSG00000198759 | EGFL6 | X | 13587724 | 13651694 | 1 | 3572.07 | 0.31 | 0.0008 | **0.0367** |
| ENSG00000196549 | MME | 3 | 154741913 | 154901497 | 1 | 5691.01 | -0.21 | 0.0008 | **0.0367** |
| ENSG00000011201 | KAL1 | X | 8496915 | 8700227 | -1 | 515.30 | -0.61 | 0.0010 | **0.0367** |
| ENSG00000180596 | HIST1H2BC | 6 | 26115101 | 26124154 | -1 | 32.28 | 0.01 | 0.0010 | **0.0367** |
| ENSG00000175449 | RFESD | 5 | 94982458 | 95020477 | 1 | 47.75 | 0.63 | 0.0012 | **0.0367** |
| ENSG00000158104 | HPD | 12 | 122277433 | 122301502 | -1 | 36.57 | 0.18 | 0.0012 | **0.0367** |
| ENSG00000230058 | ENSG00000230058 | 13 | 40918094 | 40924440 | -1 | 39.20 | -0.34 | 0.0012 | **0.0367** |
| ENSG00000178722 | C5orf64 | 5 | 60933535 | 61047590 | 1 | 51.46 | -0.11 | 0.0013 | **0.0367** |
| ENSG00000245685 | ENSG00000245685 | 4 | 190701490 | 190861426 | -1 | 158.63 | -0.21 | 0.0016 | **0.0425** |
| ENSG00000164129 | NPY5R | 4 | 164265091 | 164273086 | 1 | 469.41 | 0.18 | 0.0017 | **0.0425** |
| ENSG00000075223 | SEMA3C | 7 | 80371854 | 80551675 | -1 | 5936.05 | 0.68 | 0.0019 | **0.0430** |
| ENSG00000173114 | LRRN3 | 7 | 110731062 | 110765510 | 1 | 475.54 | -0.46 | 0.0025 | 0.0522 |
| ENSG00000060718 | COL11A1 | 1 | 103342023 | 103574052 | -1 | 149.43 | -0.06 | 0.0026 | 0.0522 |
| ENSG00000253686 | ENSG00000253686 | 5 | 173134617 | 173173214 | -1 | 75.83 | -0.50 | 0.0028 | 0.0532 |
| ENSG00000174697 | LEP | 7 | 127881337 | 127897681 | 1 | 20856.94 | -0.36 | 0.0029 | 0.0532 |
| ENSG00000077420 | APBB1IP | 10 | 26727132 | 26856732 | 1 | 1444.54 | 0.18 | 0.0038 | 0.0607 |
| ENSG00000124249 | KCNK15 | 20 | 43374421 | 43379675 | 1 | 10.33 | -0.09 | 0.0038 | 0.0607 |
| ENSG00000117682 | DHDDS | 1 | 26758773 | 26797785 | 1 | 3014.31 | -0.20 | 0.0039 | 0.0607 |
| ENSG00000153956 | CACNA2D1 | 7 | 81575760 | 82073114 | -1 | 3469.38 | 0.58 | 0.0041 | 0.0607 |
| ENSG00000185052 | SLC24A3 | 20 | 19193290 | 19703581 | 1 | 975.94 | -0.73 | 0.0043 | 0.0607 |
| ENSG00000144031 | ANKRD53 | 2 | 71205510 | 71212626 | 1 | 76.22 | -0.47 | 0.0044 | 0.0607 |
| ENSG00000260317 | ENSG00000260317 | 8 | 81453535 | 81455339 | 1 | 74.13 | 0.12 | 0.0052 | 0.0694 |
| ENSG00000164125 | FAM198B | 4 | 159045626 | 159094470 | -1 | 5014.32 | -0.02 | 0.0055 | 0.0702 |
| ENSG00000122912 | SLC25A16 | 10 | 70237756 | 70287231 | -1 | 2087.34 | -0.41 | 0.0059 | 0.0721 |
| ENSG00000206527 | PTPLB | 3 | 123209667 | 123304032 | -1 | 4348.32 | -0.61 | 0.0060 | 0.0721 |
| ENSG00000273079 | GRIN2B | 12 | 13693165 | 14133053 | -1 | 177.05 | -0.41 | 0.0068 | 0.0727 |
| ENSG00000106780 | MEGF9 | 9 | 123363091 | 123476748 | -1 | 1697.05 | 0.45 | 0.0070 | 0.0727 |
| ENSG00000164128 | NPY1R | 4 | 164245113 | 164265984 | -1 | 4618.27 | 0.31 | 0.0071 | 0.0727 |
| ENSG00000184144 | CNTN2 | 1 | 205012325 | 205047627 | 1 | 11.04 | -0.25 | 0.0071 | 0.0727 |
| ENSG00000155966 | AFF2 | X | 147582139 | 148082193 | 1 | 376.04 | -2.00 | 0.0071 | 0.0727 |
| ENSG00000173208 | ABCD2 | 12 | 39943835 | 40013553 | -1 | 1724.90 | -0.32 | 0.0075 | 0.0741 |
| ENSG00000124743 | KLHL31 | 6 | 53512699 | 53530506 | -1 | 1356.51 | -0.17 | 0.0080 | 0.0770 |
| ENSG00000099194 | SCD | 10 | 102106881 | 102124591 | 1 | 22353.39 | -0.47 | 0.0082 | 0.0770 |
| ENSG00000093144 | ECHDC1 | 6 | 127609855 | 127664754 | -1 | 3449.62 | -0.59 | 0.0086 | 0.0785 |
| ENSG00000243587 | C6orf183 | 6 | 109487036 | 109592217 | 1 | 86.54 | -0.14 | 0.0092 | 0.0821 |
| ENSG00000012660 | ELOVL5 | 6 | 53132196 | 53213947 | -1 | 10733.01 | -1.10 | 0.0101 | 0.0836 |
| ENSG00000186081 | KRT5 | 12 | 52908359 | 52914471 | -1 | 90.02 | -0.14 | 0.0101 | 0.0836 |
| ENSG00000109919 | MTCH2 | 11 | 47638867 | 47664175 | -1 | 1725.72 | -0.32 | 0.0106 | 0.0836 |
| ENSG00000154188 | ANGPT1 | 8 | 108261721 | 108510283 | -1 | 2265.87 | -0.10 | 0.0108 | 0.0836 |
| ENSG00000175267 | VWA3A | 16 | 22103859 | 22168287 | 1 | 25.51 | -0.59 | 0.0108 | 0.0836 |
| ENSG00000105971 | CAV2 | 7 | 115927434 | 116148595 | 1 | 10674.62 | -0.27 | 0.0111 | 0.0836 |
| ENSG00000134962 | KLB | 4 | 39408473 | 39453156 | 1 | 2467.31 | -0.52 | 0.0111 | 0.0836 |
| ENSG00000145757 | SPATA9 | 5 | 94987885 | 95034415 | -1 | 130.35 | 0.15 | 0.0113 | 0.0837 |
| ENSG00000113389 | NPR3 | 5 | 32689176 | 32791819 | 1 | 1387.61 | -0.28 | 0.0127 | 0.0917 |
| ENSG00000115361 | ACADL | 2 | 211052663 | 211090215 | -1 | 692.32 | -0.03 | 0.0136 | 0.0935 |
| ENSG00000147852 | VLDLR | 9 | 2621834 | 2660053 | 1 | 4310.76 | -0.06 | 0.0145 | 0.0935 |
| ENSG00000116353 | MECR | 1 | 29519385 | 29557454 | -1 | 636.82 | -0.27 | 0.0151 | 0.0935 |
| ENSG00000129682 | FGF13 | X | 137713735 | 138304939 | -1 | 434.72 | -0.45 | 0.0155 | 0.0935 |
| ENSG00000121236 | TRIM6 | 11 | 5617339 | 5634188 | 1 | 227.67 | 0.02 | 0.0158 | 0.0935 |
| ENSG00000165868 | HSPA12A | 10 | 118430703 | 118502085 | -1 | 2027.77 | -0.36 | 0.0158 | 0.0935 |
| ENSG00000170417 | TMEM182 | 2 | 103353367 | 103460352 | 1 | 210.41 | -0.49 | 0.0162 | 0.0935 |
| ENSG00000151726 | ACSL1 | 4 | 185676749 | 185747972 | -1 | 21963.94 | -1.11 | 0.0162 | 0.0935 |
| ENSG00000266968 | ENSG00000266968 | 18 | 43226118 | 43227895 | 1 | 98.11 | -0.17 | 0.0165 | 0.0935 |
| ENSG00000177614 | PGBD5 | 1 | 230457392 | 230561475 | -1 | 32.62 | 0.24 | 0.0165 | 0.0935 |
| ENSG00000067113 | PPAP2A | 5 | 54720682 | 54830878 | -1 | 2033.11 | 0.08 | 0.0165 | 0.0935 |
| ENSG00000236751 | ENSG00000236751 | X | 46185359 | 46187080 | -1 | 59.29 | -0.76 | 0.0169 | 0.0935 |
| ENSG00000141150 | RASL10B | 17 | 34058668 | 34070540 | 1 | 130.07 | -0.33 | 0.0169 | 0.0935 |
| ENSG00000065833 | ME1 | 6 | 83920108 | 84140797 | -1 | 2658.57 | -0.10 | 0.0169 | 0.0935 |
| ENSG00000151632 | AKR1C2 | 10 | 5029967 | 5060223 | -1 | 10691.58 | -0.55 | 0.0172 | 0.0935 |
| ENSG00000154269 | ENPP3 | 6 | 131949582 | 132068553 | 1 | 58.60 | -0.13 | 0.0172 | 0.0935 |
| ENSG00000169313 | P2RY12 | 3 | 151055168 | 151102600 | -1 | 207.47 | -0.22 | 0.0176 | 0.0941 |
| ENSG00000232680 | ENSG00000232680 | 19 | 35923859 | 35925544 | -1 | 10.42 | 0.09 | 0.0184 | 0.0967 |
| ENSG00000237813 | ENSG00000237813 | 7 | 115878314 | 116139519 | -1 | 29.71 | -0.55 | 0.0192 | 0.0980 |
| ENSG00000036530 | CYP46A1 | 14 | 100150641 | 100193638 | 1 | 76.18 | 0.01 | 0.0192 | 0.0980 |

Supplemental Table 4 Pathway enrichment analyses using 68 genes having differential expression between responders and non-responders

| **KEGG analyses** | | | |
| --- | --- | --- | --- |
| **Pathway name** | **Genes in set** | **Overlap** | **Adjusted pvalue** |
| Fatty acid metabolism_Homo sapiens_hsa01212 | MECR;ACADL;ELOVL5;ACSL1;SCD;PTPLB | 6 / 48 | 1.05E-06 |
| PPAR signaling pathway_Homo sapiens_hsa03320 | ACADL;ACSL1;SCD;ME1 | 4 / 69 | 0.0017 |
| Biosynthesis of unsaturated fatty acids_Homo sapiens_hsa01040 | ELOVL5;SCD;PTPLB | 3 / 23 | 0.0017 |
| Fatty acid elongation_Homo sapiens_hsa00062 | MECR;ELOVL5;PTPLB | 3 / 25 | 0.0017 |
|  |  |  |  |
| **REACTOME analyses** | | | |
| **Pathway name** | **Genes in set** | **Overlap** | **Adjusted pvalue** |
| Signaling by Leptin_Homo sapiens_R-HSA-2586552 | APBB1IP;KLB;ANGPT1;LEP;GRIN2B | 5 / 243 | 0.0448 |
| Fatty Acyl-CoA Biosynthesis_Homo sapiens_R-HSA-75105 | ELOVL5;ACSL1;SCD | 3 / 42 | 0.0409 |
| Fatty acid, triacylglycerol, and ketone body metabolism_Homo sapiens_R-HSA-535734 | ACADL;ACSL1;SCD;ELOVL5;ME1 | 5 / 217 | 0.0409 |
| Synthesis of bile acids and bile salts via 24-hydroxycholesterol_Homo sapiens_R-HSA-193775 | AKR1C2;CYP46A1 | 2 / 14 | 0.0409 |
| alpha-linolenic (omega3) and linoleic (omega6) acid metabolism_Homo sapiens_R-HSA-2046104 | ELOVL5;ACSL1 | 2 / 13 | 0.0409 |
| alpha-linolenic acid (ALA) metabolism_Homo sapiens_R-HSA-2046106 | ELOVL5;ACSL1 | 2 / 13 | 0.0409 |
| Triglyceride Biosynthesis_Homo sapiens_R-HSA-75109 | ACSL1;SCD;ELOVL5 | 3 / 66 | 0.0448 |
| Linoleic acid (LA) metabolism_Homo sapiens_R-HSA-2046105 | ELOVL5;ACSL1 | 2 / 8 | 0.0409 |

Supplemental Table 5 Results from qPCR analyses

|  |  | **Any gender** | | **Males only** | | **Females only** | |
| --- | --- | --- | --- | --- | --- | --- | --- |
|  | **gene** | **pValue** | **FDR** | **pValue** | **FDR** | **pValue** | **FDR** |
| **baseline levels** | FASN | 0.076 | 0.4557 | 0.3487 | 0.6974 | 0.0815 | 0.4182 |
|  | FADS1 | 0.4889 | 0.7334 | 0.9955 | 0.9955 | 0.3383 | 0.5370 |
|  | FADS2 | 0.9196 | 0.9305 | 0.9187 | 0.9955 | 0.8262 | 0.8262 |
|  | LEP | 0.1932 | 0.4778 | 0.8016 | 0.9955 | 0.1394 | 0.4182 |
|  | ELOVL5 | 0.2389 | 0.4778 | 0.3302 | 0.6974 | 0.3580 | 0.5370 |
|  | SCD | 0.9305 | 0.9305 | 0.3278 | 0.6974 | 0.5606 | 0.6727 |
| **changes after LCD** | FASN | 1.13E-06 | **2.26E-06** | 0.0029 | **0.0038** | 0.0001 | **0.0002** |
|  | FADS1 | 5.42E-05 | **8.13E-05** | 0.0031 | **0.0038** | 0.0844 | **0.0844** |
|  | FADS2 | 4.56E-07 | **1.37E-06** | 0.0025 | **0.0038** | 0.0003 | **0.0004** |
|  | LEP | 1.57E-04 | **1.57E-04** | 0.0004 | **0.0011** | 0.0088 | **0.0105** |
|  | ELOVL5 | 1.03E-07 | **6.17E-07** | 0.0001 | **0.0008** | 0.0003 | **0.0004** |
|  | SCD | 1.23E-04 | **1.47E-04** | 0.1537 | 0.1537 | 0.0001 | **0.0002** |
| **changes after weight maintenance** | FASN | 0.3115 | 0.3738 | 0.7581 | 0.8913 | 0.3110 | 0.3732 |
|  | FADS1 | 0.7859 | 0.7859 | 0.8913 | 0.8913 | 0.4935 | 0.4935 |
|  | FADS2 | 0.1222 | 0.1833 | 0.7094 | 0.8913 | 0.0704 | 0.1408 |
|  | LEP | 0.0048 | **0.0288** | 0.4494 | 0.8913 | 0.0011 | **0.0064** |
|  | ELOVL5 | 0.1117 | 0.1833 | 0.4555 | 0.8913 | 0.1253 | 0.1880 |
|  | SCD | 0.0367 | 0.1102 | 0.2445 | 0.8913 | 0.0505 | 0.1408 |

Supplemental Table 6 Proteomics results:  somamers whose baseline levels significantly associate with the lipid signature

| **SOMAMER** | **TARGET** | **ENTREZ GENE SYMBOL** | **ENTREZ GENE ID** | **UNIPROT** | **pValue** | **FDR** |
| --- | --- | --- | --- | --- | --- | --- |
| SL000276 | Apo E | APOE | 348 | P02649 | 3.10E-07 | 2.0E-4 |
| SL004668 | Apo E3 | APOE | 348 | P02649 | 3.64E-07 | 20E-4 |
| SL004669 | Apo E4 | APOE | 348 | P02649 | 7.44E-07 | 2.7E-4 |
| SL000277 | Apo E2 | APOE | 348 | P02649 | 5.10E-05 | 0.0144 |

Supplemental Table 7 Proteomics results : somamers whose change in levels during LCD significantly associate with the lipid signature

| **SOMAMER** | **TARGET** | **ENTREZ GENE SYMBOL** | **ENTREZ GENE ID** | **UNIPROT** | **Regression Coefficient** | **Standard Error** | **pValue** | **FDR** |
| --- | --- | --- | --- | --- | --- | --- | --- | --- |
| SL006397 | NRP1 | NRP1 | 8829 | O14786 | 2.09 | 0.27 | 1.48E-13 | 1.67E-10 |
| SL000276 | Apo E | APOE | 348 | P02649 | -3.19 | 0.54 | 9.49E-09 | 3.29E-06 |
| SL004669 | Apo E4 | APOE | 348 | P02649 | -2.54 | 0.43 | 8.59E-09 | 3.29E-06 |
| SL000249 | a1-Antitrypsin | SERPINA1 | 5265 | P01009 | 1.98 | 0.34 | 1.46E-08 | 3.29E-06 |
| SL005102 | SHBG | SHBG | 6462 | P04278 | 3.11 | 0.53 | 1.32E-08 | 3.29E-06 |
| SL003197 | TECK | CCL25 | 6370 | O15444 | -3.77 | 0.65 | 2.18E-08 | 4.10E-06 |
| SL004588 | IL-1 R AcP | IL1RAP | 3556 | Q9NPH3 | 1.55 | 0.27 | 2.91E-08 | 4.69E-06 |
| SL004660 | BSP | IBSP | 3381 | P21815 | 2.38 | 0.43 | 6.31E-08 | 8.90E-06 |
| SL001996 | Angiopoietin-2 | ANGPT2 | 285 | O15123 | 2.02 | 0.37 | 1.62E-07 | 1.82E-05 |
| SL000358 | Coagulation Factor VII | F7 | 2155 | P08709 | -1.37 | 0.25 | 1.60E-07 | 1.82E-05 |
| SL004668 | Apo E3 | APOE | 348 | P02649 | -2.14 | 0.40 | 2.38E-07 | 2.45E-05 |
| SL009045 | ENPP7 | ENPP7 | 339221 | Q6UWV6 | -3.43 | 0.66 | 4.69E-07 | 4.42E-05 |
| SL000466 | IGFBP-2 | IGFBP2 | 3485 | P18065 | 2.64 | 0.51 | 5.10E-07 | 4.43E-05 |
| SL004782 | TSG-6 | TNFAIP6 | 7130 | P98066 | 2.25 | 0.44 | 7.80E-07 | 6.29E-05 |
| SL000325 | C9 | C9 | 735 | P02748 | 1.44 | 0.28 | 8.60E-07 | 6.47E-05 |
| SL004143 | GFRa-2 | GFRA2 | 2675 | O00451 | 1.30 | 0.26 | 1.06E-06 | 7.48E-05 |
| SL000019 | Apo A-I | APOA1 | 335 | P02647 | -1.68 | 0.34 | 1.69E-06 | 0.00011 |
| SL000440 | Hemopexin | HPX | 3263 | P02790 | 1.79 | 0.37 | 1.87E-06 | 0.00012 |
| SL001998 | TFPI | TFPI | 7035 | P10646 | -1.24 | 0.26 | 2.17E-06 | 0.00013 |
| SL004672 | BCMA | TNFRSF17 | 608 | Q02223 | 1.35 | 0.28 | 2.52E-06 | 0.00014 |
| SL001774 | FABP | FABP3 | 2170 | P05413 | 2.00 | 0.41 | 2.62E-06 | 0.00014 |
| SL005168 | Growth hormone receptor | GHR | 2690 | P10912 | -1.86 | 0.39 | 3.05E-06 | 0.00016 |
| SL005152 | TIG2 | RARRES2 | 5919 | Q99969 | -1.38 | 0.31 | 1.17E-05 | 0.00057 |
| SL003994 | BMP-1 | BMP1 | 649 | P13497 | -1.83 | 0.41 | 1.52E-05 | 0.00072 |
| SL000053 | tPA | PLAT | 5327 | P00750 | -1.95 | 0.45 | 2.16E-05 | 0.00094 |
| SL000360 | Coagulation Factor X | F10 | 2159 | P00742 | -0.81 | 0.19 | 2.11E-05 | 0.00094 |
| SL000277 | Apo E2 | APOE | 348 | P02649 | -1.43 | 0.33 | 2.60E-05 | 0.00109 |
| SL007547 | TIMD3 | HAVCR2 | 84868 | Q8TDQ0 | 1.64 | 0.39 | 3.63E-05 | 0.00146 |
| SL003322 | VEGF sR3 | FLT4 | 2324 | P35916 | 1.17 | 0.28 | 4.04E-05 | 0.00157 |
| SL007429 | GPNMB | GPNMB | 10457 | Q14956 | 1.45 | 0.36 | 7.81E-05 | 0.00294 |
| SL004639 | TrkC | NTRK3 | 4916 | Q16288 | 1.55 | 0.39 | 9.07E-05 | 0.00323 |
| SL000541 | Plasminogen | PLG | 5340 | P00747 | -0.88 | 0.22 | 9.14E-05 | 0.00323 |
| SL004118 | TrATPase | ACP5 | 54 | P13686 | -1.17 | 0.30 | 0.00010 | 0.00343 |
| SL004466 | Heparin cofactor II | SERPIND1 | 3053 | P05546 | -0.85 | 0.22 | 0.00010 | 0.00343 |
| SL004712 | SDF-1 | CXCL12 | 6387 | P48061 | 1.22 | 0.31 | 0.00011 | 0.00358 |
| SL012707 | PCSK9 | PCSK9 | 255738 | Q8NBP7 | -1.49 | 0.38 | 0.00011 | 0.00358 |
| SL003060 | bFGF-R | FGFR1 | 2260 | P11362 | 1.03 | 0.26 | 0.00013 | 0.00401 |
| SL017613 | FCG2A/B | FCGR2A FCGR2B | None | P12318 P31994 | 1.40 | 0.37 | 0.00016 | 0.00484 |
| SL008639 | IDS | IDS | 3423 | P22304 | 2.28 | 0.60 | 0.00017 | 0.00502 |
| SL010458 | Endocan | ESM1 | 11082 | Q9NQ30 | 1.07 | 0.28 | 0.00019 | 0.00519 |
| SL010471 | Testican-2 | SPOCK2 | 9806 | Q92563 | 0.98 | 0.26 | 0.00019 | 0.00519 |
| SL003764 | NCAM-120 | NCAM1 | 4684 | P13591 | 0.92 | 0.25 | 0.00022 | 0.00584 |
| SL009213 | Cathepsin A | CTSA | 5476 | P10619 | -1.94 | 0.52 | 0.00024 | 0.00624 |
| SL006460 | GP1BA | GP1BA | 2811 | P07359 | 0.95 | 0.25 | 0.00025 | 0.00627 |
| SL005846 | Moesin | MSN | 4478 | P26038 | -1.53 | 0.41 | 0.00025 | 0.00627 |
| SL000382 | CK-MB | CKB CKM | 1152 1158 | P12277 P06732 | 2.90 | 0.79 | 0.00032 | 0.00793 |
| SL000134 | Met | MET | 4233 | P08581 | 0.92 | 0.26 | 0.00043 | 0.01000 |
| SL004718 | Karyopherin-a2 | KPNA2 | 3838 | P52292 | 1.07 | 0.30 | 0.00043 | 0.01000 |
| SL003324 | Coagulation Factor Xa | F10 | 2159 | P00742 | -0.78 | 0.22 | 0.00043 | 0.01000 |
| SL007642 | ANGL4 | ANGPTL4 | 51129 | Q9BY76 | 1.39 | 0.39 | 0.00048 | 0.01079 |
| SL000006 | PAI-1 | SERPINE1 | 5054 | P05121 | -3.35 | 0.95 | 0.00050 | 0.01082 |
| SL007674 | LY9 | LY9 | 4063 | Q9HBG7 | 1.59 | 0.45 | 0.00049 | 0.01082 |
| SL008904 | LYVE1 | LYVE1 | 10894 | Q9Y5Y7 | 1.51 | 0.43 | 0.00052 | 0.01102 |
| SL000507 | Lymphotoxin a1/b2 | LTA LTB | 4049 4050 | P01374 Q06643 | 1.00 | 0.29 | 0.00055 | 0.01144 |
| SL004855 | contactin-1 | CNTN1 | 1272 | Q12860 | 0.90 | 0.26 | 0.00064 | 0.01315 |
| SL003679 | IGF-II receptor | IGF2R | 3482 | P11717 | -0.97 | 0.28 | 0.00065 | 0.01315 |
| SL008644 | BST1 | BST1 | 683 | Q10588 | 1.45 | 0.42 | 0.00066 | 0.01315 |
| SL005208 | Nogo Receptor | RTN4R | 65078 | Q9BZR6 | -0.97 | 0.28 | 0.00071 | 0.01381 |
| SL005574 | Aminoacylase-1 | ACY1 | 95 | Q03154 | -3.01 | 0.88 | 0.00073 | 0.01398 |
| SL000091 | PSA-ACT | KLK3 SERPINA3 | 354 12 | P07288 P01011 | 1.13 | 0.33 | 0.00074 | 0.01398 |
| SL000248 | a1-Antichymotrypsin | SERPINA3 | 12 | P01011 | 0.89 | 0.26 | 0.00081 | 0.01493 |
| SL004482 | Endoglin | ENG | 2022 | P17813 | 1.30 | 0.39 | 0.00089 | 0.01627 |
| SL004635 | CD30 Ligand | TNFSF8 | 944 | P32971 | 0.87 | 0.26 | 0.00094 | 0.01689 |
| SL000462 | IGFBP-1 | IGFBP1 | 3484 | P08833 | 2.80 | 0.84 | 0.00104 | 0.01783 |
| SL005797 | PIGR | PIGR | 5284 | P01833 | -1.64 | 0.49 | 0.00104 | 0.01783 |
| SL004661 | Aggrecan | ACAN | 176 | P16112 | 1.02 | 0.31 | 0.00103 | 0.01783 |
| SL005263 | RAP | LRPAP1 | 4043 | P30533 | -1.34 | 0.41 | 0.00110 | 0.01850 |
| SL000272 | Antithrombin III | SERPINC1 | 462 | P01008 | 0.51 | 0.16 | 0.00113 | 0.01875 |
| SL009089 | PGCB | BCAN | 63827 | Q96GW7 | 0.89 | 0.27 | 0.00120 | 0.01958 |
| SL002506 | suPAR | PLAUR | 5329 | Q03405 | 1.17 | 0.36 | 0.00122 | 0.01974 |
| SL000498 | Leptin | LEP | 3952 | P41159 | -2.35 | 0.72 | 0.00127 | 0.02009 |
| SL002086 | Ficolin-3 | FCN3 | 8547 | O75636 | -1.10 | 0.34 | 0.00128 | 0.02009 |
| SL000573 | SAP | APCS | 325 | P02743 | -0.89 | 0.28 | 0.00138 | 0.02140 |
| SL000592 | TIMP-2 | TIMP2 | 7077 | P16035 | 0.88 | 0.27 | 0.00143 | 0.02175 |
| SL000383 | CK-MM | CKM | 1158 | P06732 | 1.94 | 0.60 | 0.00145 | 0.02189 |
| SL014268 | OX2G | CD200 | 4345 | P41217 | 1.04 | 0.33 | 0.00176 | 0.02611 |
| SL010378 | RET | RET | 5979 | P07949 | -1.20 | 0.38 | 0.00181 | 0.02653 |
| SL003329 | HCC-1 | CCL14 | 6358 | Q16627 | 1.27 | 0.40 | 0.00184 | 0.02668 |
| SL005215 | Siglec-3 | CD33 | 945 | P20138 | 1.63 | 0.52 | 0.00190 | 0.02715 |
| SL008773 | CD109 | CD109 | 135228 | Q6YHK3 | 1.17 | 0.37 | 0.00202 | 0.02857 |
| SL005223 | TCCR | IL27RA | 9466 | Q6UWB1 | 0.84 | 0.27 | 0.00214 | 0.02986 |
| SL004739 | ITI heavy chain H4 | ITIH4 | 3700 | Q14624 | 0.88 | 0.29 | 0.00234 | 0.03182 |
| SL014292 | SIG14 | SIGLEC14 | 100049587 | Q08ET2 | 1.10 | 0.36 | 0.00232 | 0.03182 |
| SL004064 | GIB | PLA2G1B | 5319 | P04054 | 1.12 | 0.37 | 0.00246 | 0.03306 |
| SL005196 | LSAMP | LSAMP | 4045 | Q13449 | 0.66 | 0.21 | 0.00252 | 0.03343 |
| SL004080 | BMPR1A | BMPR1A | 657 | P36894 | 1.11 | 0.37 | 0.00258 | 0.03382 |
| SL000678 | Granulysin | GNLY | 10578 | P22749 | 0.99 | 0.33 | 0.00272 | 0.03504 |
| SL004876 | Kallistatin | SERPINA4 | 5267 | P29622 | -0.56 | 0.19 | 0.00273 | 0.03504 |
| SL003328 | Factor I | CFI | 3426 | P05156 | -0.54 | 0.18 | 0.00293 | 0.03514 |
| SL000308 | C1-Esterase Inhibitor | SERPING1 | 710 | P05155 | 1.10 | 0.37 | 0.00289 | 0.03514 |
| SL013969 | KYNU | KYNU | 8942 | Q16719 | -1.42 | 0.47 | 0.00291 | 0.03514 |
| SL002823 | sL-Selectin | SELL | 6402 | P14151 | 0.77 | 0.26 | 0.00285 | 0.03514 |
| SL004863 | TAJ | TNFRSF19 | 55504 | Q9NS68 | 0.90 | 0.30 | 0.00292 | 0.03514 |
| SL003184 | sLeptin R | LEPR | 3953 | P48357 | 1.12 | 0.37 | 0.00289 | 0.03514 |
| SL002763 | Kallikrein 11 | KLK11 | 11012 | Q9UBX7 | 1.08 | 0.36 | 0.00322 | 0.03828 |
| SL011809 | XTP3A | DCTPP1 | 79077 | Q9H773 | 1.10 | 0.37 | 0.00327 | 0.03850 |
| SL009412 | DKK3 | DKK3 | 27122 | Q9UBP4 | 0.67 | 0.23 | 0.00334 | 0.03886 |
| SL003200 | sTie-2 | TEK | 7010 | Q02763 | 0.70 | 0.24 | 0.00338 | 0.03889 |
| SL010368 | IDUA | IDUA | 3425 | P35475 | -0.93 | 0.32 | 0.00346 | 0.03947 |
| SL008574 | OMD | OMD | 4958 | Q99983 | 1.19 | 0.40 | 0.00350 | 0.03954 |
| SL000268 | Angiostatin | PLG | 5340 | P00747 | -0.85 | 0.29 | 0.00359 | 0.04013 |
| SL004652 | WIF-1 | WIF1 | 11197 | Q9Y5W5 | 0.88 | 0.30 | 0.00368 | 0.04069 |
| SL005205 | NKp30 | NCR3 | 259197 | O14931 | 0.57 | 0.20 | 0.00386 | 0.04232 |
| SL014070 | SLIK5 | SLITRK5 | 26050 | O94991 | 1.04 | 0.36 | 0.00397 | 0.04307 |
| SL003300 | HCC-4 | CCL16 | 6360 | O15467 | -1.10 | 0.38 | 0.00426 | 0.04585 |
| SL016928 | SLAF7 | SLAMF7 | 57823 | Q9NQ25 | 1.24 | 0.43 | 0.00431 | 0.04586 |
| SL014008 | FUT5 | FUT5 | 2527 | Q11128 | 1.50 | 0.52 | 0.00454 | 0.04739 |
| SL000021 | Insulin | INS | 3630 | P01308 | -1.01 | 0.35 | 0.00457 | 0.04739 |
| SL005190 | ILT-2 | LILRB1 | 10859 | Q8NHL6 | 0.72 | 0.25 | 0.00451 | 0.04739 |
| SL000516 | MCP-3 | CCL7 | 6354 | P80098 | 0.90 | 0.31 | 0.00475 | 0.04878 |
| SL007195 | CD70 | CD70 | 970 | P32970 | -0.71 | 0.25 | 0.00483 | 0.04908 |

Supplemental Table 8 Pathway enrichment analyses using 106 proteins having differential fold-change between responders and non-responders

| **KEGG analyses** | | | |
| --- | --- | --- | --- |
| Pathway name | Proteins In Set | Overlap | Adjusted pvalue |
| COMPLEMENT_AND_COAGULATION_CASCADES | PLAT, CFI, PLAUR, SERPINE1, C9, F10, F7, SERPIND1, TFPI, SERPINC1, SERPINA1, PLG, SERPING1 | 13 / 33 | 1.25E-04 |
| ARGININE_AND_PROLINE_METABOLISM | CKM, ACY1, CKB | 3 / 2 | 0.0049 |
| LYSOSOME | IDUA, CTSA, IDS, ACP5, IGF2R | 5 / 14 | 0.0215 |
| NEUROACTIVE_LIGAND_RECEPTOR_INTERACTION | LEP, GHR, PLG, LEPR | 4 / 10 | 0.0262 |
| PPAR_SIGNALING_PATHWAY | APOA1, ANGPTL4, FABP3 | 3 / 6 | 0.0303 |
| GLYCOSAMINOGLYCAN_DEGRADATION | IDUA, IDS | 2 / 2 | 0.0316 |
|  |  |  |  |
| **REACTOME analyses** | | | |
| Pathway name | Proteins In Set | Overlap | Adjusted pvalue |
| GLYCOSAMINOGLYCAN_METABOLISM | IDUA, IDS, LYVE1, ACAN, BCAN, OMD | 6 / 12 | 0.0036 |
| FORMATION_OF_FIBRIN_CLOT_CLOTTING_CASCADE | F10, F7, TFPI, SERPINC1, SERPING1, GP1BA | 6 / 15 | 0.0083 |
| CS_DS_DEGRADATION | IDUA, IDS, BCAN | 3 / 3 | 0.0090 |
| HDL_MEDIATED_LIPID_TRANSPORT | APOE, APOA1, BMP1 | 3 / 3 | 0.0090 |
| DIABETES_PATHWAYS | IGFBP2, LEP, IGFBP1, PLG, KLK3, INS | 6 / 18 | 0.0147 |
| REGULATION_OF_INSULIN_LIKE_GROWTH_FACTOR_IGF_ACTIVITY_BY_INSULIN_LIKE_GROWTH_FACTOR_BINDING_PROTEINS_IGFBPS | IGFBP2, IGFBP1, PLG, KLK3 | 4 / 9 | 0.0190 |
| LIPOPROTEIN_METABOLISM | APOE, APOA1, BMP1 | 3 / 5 | 0.0215 |
| HEPARAN_SULFATE_HEPARIN_HS_GAG_METABOLISM | IDUA, IDS, BCAN | 3 / 6 | 0.0295 |
| LIPID_DIGESTION_MOBILIZATION_AND_TRANSPORT | APOE, APOA1, BMP1 | 3 / 6 | 0.0295 |
| CHYLOMICRON_MEDIATED_LIPID_TRANSPORT | APOE, APOA1 | 2 / 2 | 0.0342 |
| KERATAN_SULFATE_BIOSYNTHESIS | ACAN, OMD | 2 / 2 | 0.0342 |
| SYNTHESIS_SECRETION_AND_DEACYLATION_OF_GHRELIN | LEP, INS | 2 / 2 | 0.0342 |
| METABOLISM_OF_CARBOHYDRATES | IDUA, IDS, LYVE1, ACAN, BCAN, OMD | 6 / 24 | 0.0486 |
| CHONDROITIN_SULFATE_DERMATAN_SULFATE_METABOLISM | IDUA, IDS, BCAN | 3 / 7 | 0.0495 |
|  |  |  |  |
| **PANTHER analyses** | | | |
| Pathway name | Proteins In Set | Overlap | Adjusted pvalue |
| BLOOD COAGULATION | PLAT, PLAUR, SERPINE1, F10, F7, TFPI, SERPINC1, SERPINA1, PLG, GP1BA | 10 / 14 | 5.81E-05 |
| PLASMINOGEN ACTIVATING CASCADE | PLAT, PLAUR, SERPINE1, PLG | 4 / 9 | 0.0219 |
